# Effects of Psilocybin-Assisted Therapy on Major Depressive Disorder: A Randomized Clinical Trial

**DOI:** 10.1001/jamapsychiatry.2020.3285

**Published:** 2020-11-04

**Authors:** Alan K. Davis, Frederick S. Barrett, Darrick G. May, Mary P. Cosimano, Nathan D. Sepeda, Matthew W. Johnson, Patrick H. Finan, Roland R. Griffiths

**Affiliations:** 1 Center for Psychedelic and Consciousness Research, Department of Psychiatry and Behavioral Sciences, Johns Hopkins School of Medicine, Baltimore, Maryland; 2 College of Social Work, The Ohio State University, Columbus; 3 Department of Neuroscience, Johns Hopkins School of Medicine, Baltimore, Maryland

## Abstract

**Importance:**

Major depressive disorder (MDD) is a substantial public health burden, but current treatments have limited effectiveness and adherence. Recent evidence suggests that 1 or 2 administrations of psilocybin with psychological support produces antidepressant effects in patients with cancer and in those with treatment-resistant depression.

**Objective:**

To investigate the effect of psilocybin therapy in patients with MDD.

**Design, Setting, and Participants:**

This randomized, waiting list–controlled clinical trial was conducted at the Center for Psychedelic and Consciousness Research at Johns Hopkins Bayview Medical Center in Baltimore, Maryland. Adults aged 21 to 75 years with an MDD diagnosis, not currently using antidepressant medications, and without histories of psychotic disorder, serious suicide attempt, or hospitalization were eligible to participate. Enrollment occurred between August 2017 and April 2019, and the 4-week primary outcome assessments were completed in July 2019. A total of 27 participants were randomized to an immediate treatment condition group (n = 15) or delayed treatment condition group (waiting list control condition; n = 12). Data analysis was conducted from July 1, 2019, to July 31, 2020, and included participants who completed the intervention (evaluable population).

**Interventions:**

Two psilocybin sessions (session 1: 20 mg/70 kg; session 2: 30 mg/70 kg) were given (administered in opaque gelatin capsules with approximately 100 mL of water) in the context of supportive psychotherapy (approximately 11 hours). Participants were randomized to begin treatment immediately or after an 8-week delay.

**Main Outcomes and Measures:**

The primary outcome, depression severity was assessed with the GRID-Hamilton Depression Rating Scale (GRID-HAMD) scores at baseline (score of ≥17 required for enrollment) and weeks 5 and 8 after enrollment for the delayed treatment group, which corresponded to weeks 1 and 4 after the intervention for the immediate treatment group. Secondary outcomes included the Quick Inventory of Depressive Symptomatology-Self Rated (QIDS-SR).

**Results:**

Of the randomized participants, 24 of 27 (89%) completed the intervention and the week 1 and week 4 postsession assessments. This population had a mean (SD) age of 39.8 (12.2) years, was composed of 16 women (67%), and had a mean (SD) baseline GRID-HAMD score of 22.8 (3.9). The mean (SD) GRID-HAMD scores at weeks 1 and 4 (8.0 [7.1] and 8.5 [5.7]) in the immediate treatment group were statistically significantly lower than the scores at the comparable time points of weeks 5 and 8 (23.8 [5.4] and 23.5 [6.0]) in the delayed treatment group. The effect sizes were large at week 5 (Cohen *d* = 2.5; 95% CI, 1.4-3.5; *P* < .001) and week 8 (Cohen *d* = 2.6; 95% CI, 1.5-3.7; *P* < .001). The QIDS-SR documented a rapid decrease in mean (SD) depression score from baseline to day 1 after session 1 (16.7 [3.5] vs 6.3 [4.4]; Cohen *d* = 2.6; 95% CI, 1.8-3.5; *P* < .001), which remained statistically significantly reduced through the week 4 follow-up (6.0 [5.7]; Cohen *d* = 2.3; 95% CI, 1.5-3.0; *P* < .001). In the overall sample, 17 participants (71%) at week 1 and 17 (71%) at week 4 had a clinically significant response to the intervention (≥50% reduction in GRID-HAMD score), and 14 participants (58%) at week 1 and 13 participants (54%) at week 4 were in remission (≤7 GRID-HAMD score).

**Conclusions and Relevance:**

Findings suggest that psilocybin with therapy is efficacious in treating MDD, thus extending the results of previous studies of this intervention in patients with cancer and depression and of a nonrandomized study in patients with treatment-resistant depression.

**Trial Registration:**

ClinicalTrials.gov Identifier: NCT03181529

## Key Points

**Question:**

Is psilocybin-assisted therapy efficacious among patients with major depressive disorder?

**Findings:**

In this randomized clinical trial of 24 participants with major depressive disorder, participants who received immediate psilocybin-assisted therapy compared with delayed treatment showed improvement in blinded clinician rater–assessed depression severity and in self-reported secondary outcomes through the 1-month follow-up.

**Meaning:**

This randomized clinical trial found that psilocybin-assisted therapy was efficacious in producing large, rapid, and sustained antidepressant effects in patients with major depressive disorder.

## Teaser

This randomized clinical trial examines the efficacy of psilocybin as an adjunct to psychotherapy and other treatments for major depressive disorder.

## Introduction

Major depressive disorder (MDD) is a substantial public health concern, affecting more than 300 million individuals worldwide. Depression is the number one cause of disability,^1^ and the relative risk of all-cause mortality for those with depression is 1.7 times greater than the risk for the general public.^2^ In the United States, approximately 10% of the adult population has been diagnosed with MDD in the past 12 months,^3^ and the yearly economic burden of MDD is estimated to be $210 billion.^4^

Although effective pharmacotherapies for depression are available, these drugs have limited efficacy, produce adverse effects, and are associated with patient adherence problems.^5^ Although many patients with depression showed reduced or remitted symptoms after treatment with existing pharmacotherapies,^6^ approximately 30% to 50% of patients did not respond fully and as many as 10% to 30% of patients were considered treatment-resistant, resulting in average effects that were only modestly larger than the effects of placebo.^7,8^

Most of the current pharmacotherapies for MDD, including the widely used selective serotonin reuptake inhibitors, increase levels of brain monoamine neurotransmitters such as serotonin and norepinephrine (typically by blocking reuptake).^6^ A growing body of evidence suggests that newer ketamine-like medications exert therapeutic efficacy in MDD through effects on glutamate neurotransmission.^9,10^ Ketamine hydrochloride, a nonselective *N*-methyl-d-aspartate receptor antagonist, is the most well-researched of these newer medications. Several studies have demonstrated the efficacy of a single ketamine infusion in rapidly (within hours) reducing depression symptoms and, when effective, lasting from a few days to about 2 weeks.^10,11^ However, ketamine has high abuse liability, and its administration involves moderate physiological risk that requires medical monitoring.^12^

The combined serotonergic and glutamatergic action of psilocybin^13,14,15^ (a classic hallucinogen) and the preliminary evidence of the antidepressant effects of psilocybin-assisted therapy (among patients with life-threatening cancer or patients with treatment-resistant depression)^16,17,18^ indicate the potential of psilocybin-assisted therapy as a novel antidepressant intervention.^19^ Moreover, psilocybin has lower addiction liability and toxic effects compared with ketamine^20,21,22^ and is generally not associated with long-term perceptual, cognitive, or neurological dysfunction.^23^

The substantial negative public health impact of MDD underscores the importance of conducting more research into drugs with rapid and sustained antidepressant effects. Current pharmacotherapies for depression have variable efficacy and unwanted adverse effects. Novel antidepressants with rapid and sustained effects on mood and cognition could represent a breakthrough in the treatment of depression and may potentially improve or save lives. Therefore, the primary objective of this randomized clinical trial was to investigate the effect of psilocybin therapy in patients with MDD.

## Method

This randomized, waiting list–controlled clinical trial was conducted at the Center for Psychedelic and Consciousness Research in Baltimore, Maryland. The Johns Hopkins Medicine Institutional Review Board approved this trial (the protocol is included in Supplement 1). Written informed consent was obtained from all participants.

### Study Design and Participants

This trial of psilocybin therapy included participants with moderate or severe MDD episodes, as assessed with the Structured Clinical Interview for DSM-5 (SCID-5)^24^ and the GRID-Hamilton Depression Rating Scale (GRID-HAMD; a score of ≥17 was required for enrollment).^25,26^ Eligible candidates were aged 21 to 75 years who self-reported no current pharmacotherapy for depression at trial screening. To avoid the confounding effects and potential interactions of concurrent antidepressant use, candidates were required to refrain from using antidepressants (eg, selective serotonin reuptake inhibitors) for at least 5 half-lives before the screening and up to 4 months after enrollment (through the completion of the primary outcome assessment). However, the decision to taper off and/or continuing not to take their medications during the study was made by the individuals and their prescribing physicians and not by study personnel. Additional eligibility requirements included being medically stable with no uncontrolled cardiovascular conditions; having no personal or family history (first or second degree) of psychotic or bipolar disorders; and, for women, being nonpregnant, being non-nursing, and agreeing to use contraception. Individuals with a moderate or severe alcohol or other drug use disorder (including nicotine) in the past year, as defined by *Diagnostic and Statistical Manual of Mental Disorders* (Fifth Edition) (*DSM-5*) criteria, were excluded, as were individuals with substantial lifetime use (>10 total) or recent use (past 6 months) of ketamine or classic hallucinogens, such as psilocybin-containing mushrooms or lysergic acid diethylamide (eMethods in Supplement 2).

**Figure 1.**
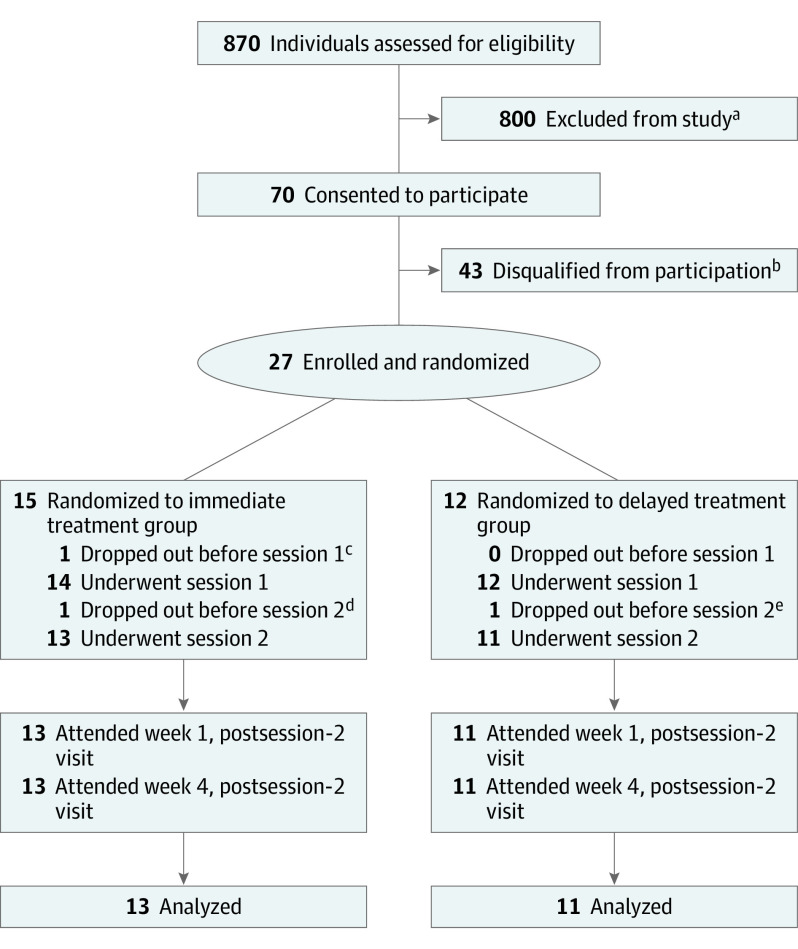
CONSORT Diagram of Participant Flow ^a^After completing the prescreening questionnaire, people were deemed ineligible if they were currently using antidepressant medication (n = 157); lived outside reasonable commuting distance (n = 161); did not meet criteria for the magnetic resonance imaging scans (n = 99); had a first- or second-degree relative with a diagnosis of schizophrenia spectrum, bipolar I or II, or other psychotic disorder ( = 77); had a recent history of substance use disorder (n = 50); opted out of in-person screening (n = 38); were not in a current depressive episode (n = 37); were more than 25% beyond the upper or lower range of recommended body weight (n = 32); had a medically significant suicide attempt (n = 30); had lifetime hallucinogen use that exceeded the exclusion threshold (n = 30); if major depressive disorder (MDD) was not primary psychiatric diagnosis (n = 18); if they had a medical exclusion (n = 11); had exclusionary use of nonserotonergic psychoactive medication (n = 11); or failed to respond to electroconvulsive therapy during current depressive episode (n = 4). Forty-five people were ineligible for other reasons. ^b^People were deemed ineligible during in-person screening if they had a psychiatric condition judged to be incompatible with establishment of rapport or safe exposure to psilocybin (n = 17); did not have confirmed *DSM-5* diagnosis of MDD (n = 7); had a recent history of moderate to severe substance use disorder (n = 5); were at high risk for suicidality (n = 3); disagreed with study procedures (n = 3); had a baseline GRID Hamilton Depression Rating Scale score lower than the eligibility threshold of 17 (n = 2); had cardiovascular conditions (n = 2); had lifetime hallucinogen use that exceeded the exclusion threshold (n = 2); were currently taking serotonergic medication (n = 1); or were more than 25% beyond the upper and lower range of recommended body weight (n = 1). ^c^Dropped out of the study due to anticipatory anxiety about the upcoming first psilocybin session. ^d^Dropped out of study due to sleep difficulties. Sleep difficulties were also reported at screening, and it was not clear whether sleep difficulties were exacerbated by the intervention. ^e^Participant showed a marked reduction in depression symptoms immediately following the first psilocybin session and chose not to proceed with the intervention.

Participants were enrolled between August 2017 and April 2019, and the 4-week primary outcome assessments were completed in July 2019. Recruitment was carried out through flyers, print advertisements, internet forums, social media, and the study website. Of the 870 individuals screened by telephone or electronic screening survey, 70 went on to undergo in-person medical and psychological screening, 43 were disqualified, and 27 qualified and were enrolled in the study. After screening, baseline assessments, and enrollment, 27 participants were randomized to either the immediate treatment group or the delayed treatment group (ie, the waiting list control condition). The use of a delayed treatment control was chosen to differentiate the psilocybin intervention from spontaneous symptom improvement. The delay interval was 8 weeks, after which participants in the delayed treatment group underwent all study assessments and entered the study intervention period. Randomization to the immediate treatment and delayed treatment groups occurred after screening and baseline assessments (Figure 1). Participants were randomized using urn randomization,^27^ balancing for sex, age, depression severity at screening (assessed using the GRID-HAMD), and level of treatment resistance (assessed using the Maudsley Staging Method).^28^ One of us (F.S.B.), who was not involved in participant screening or enrollment, performed urn randomization using the randPack library, version 1.32.0,^29^ in the R Statistical Software package (R Foundation for Statistical Computing).^30^

Participants received no monetary compensation for undergoing the intervention. However, participants received a total of $200 for completing 2 magnetic resonance imaging sessions.

### Immediate Treatment Condition

**Figure 2.**
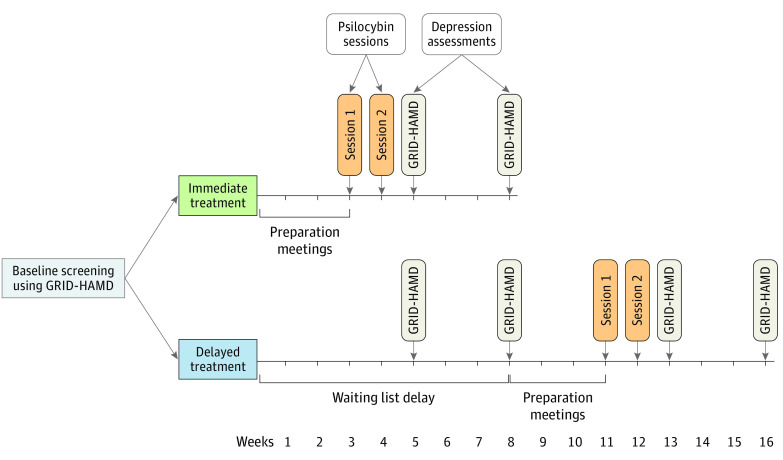
Study Timeline From Baseline Assessment and Screening to the 4-Week Postsession-2 Follow-up Visit GRID-HAMD indicates GRID Hamilton Depression Rating Scale.

The intervention period was 8 weeks and involved at least 18 in-person visits, including 2 daylong psilocybin administration sessions (Figure 2). Consistent with previous studies using psilocybin,^16,31^ the visit schedule included preparatory meetings (8 hours in total) with 2 session facilitators before the first psilocybin session as well as follow-up meetings after psilocybin sessions (2-3 hours in total) (eMethods in Supplement 2). Session facilitators were study staff with varying educational levels (ie, bachelor’s, master’s, doctorate, and medical degrees) and professional disciplines (eg, social work, psychology, and psychiatry). After the preparation meetings, 2 psilocybin administration sessions were conducted a mean of 1.6 weeks apart (no statistically significant differences were found between conditions; eResults in Supplement 2). The psilocybin dose was moderately high (20 mg/70 kg) in session 1 and was high (30 mg/70 kg) in session 2. Procedures for psilocybin administration and the conduct of the sessions were similar to procedures used in previous and ongoing studies with psilocybin (eMethods in Supplement 2) at the Center for Psychedelic and Consciousness Research.^16,32,33^

Psilocybin was administered in opaque gelatin capsules with approximately 100 mL water. Both facilitators were present in the room and available to respond to participants’ physical and emotional needs during the day-long session, with the exception of short breaks taken by 1 facilitator at a time. During the session, participants were instructed to lie on a couch in a living room–like environment, and facilitators encouraged participants to focus their attention inward and stay with any experience that arose. To enhance inward reflection, music was played (the playlist is provided in the eMethods in Supplement 2), and participants were instructed to wear eyeshades and headphones.

### Delayed Treatment Condition

For safety during the 8-week delay period of the delayed treatment group, participants were monitored weekly by in-person assessment or brief telephone calls. In weeks 5 and 8, participants attended an in-person visit and underwent the GRID-HAMD assessment and other study measures. In other weeks of the delay period, participants received telephone calls that included a brief check-in and assessment for self-reported suicidal ideation or behavior and depression symptoms. All assessments during the delay period were administered by study staff who were not lead facilitators. At the end of the delay period, all participants in the delayed treatment group completed the same intervention as the participants in the immediate treatment group.

### Outcome Assessments

Screening evaluation included a preliminary questionnaire administered via telephone or an online survey as well as an in-person medical history and physical examination, electrocardiogram, routine medical blood and urinalysis laboratory tests, and structured assessments (eg, SCID-5, SCID-5 Screening Personality Questionnaire, SCID-5 Personality Disorders, and Personality Assessment Inventory).^24,34,35,36^

The primary outcome measure was the GRID-HAMD,^37^ a version of the 17-item Hamilton Depression Rating Scale that has high reliability and validity.^26^ The GRID-HAMD was administered by blinded clinician raters via telephone at baseline and at postrandomization weeks 5 and 8 for participants in the delayed treatment group and at the weeks 1 and 4 follow-up visits after the second psilocybin session for participants in both the immediate treatment and delayed treatment groups. The primary between-group end point comparison was at weeks 5 and 8 between the immediate treatment and delayed treatment groups (Figure 2). The primary within-group end point comparison was between baseline and weeks 1 and 4 postsession 2 follow-up visits in both groups.

Severity of depression was assessed using the total GRID-HAMD score (0-7: no depression; 8-16: mild depression; 17-23: moderate depression; ≥24: severe depression).^38^ A clinically significant response was defined as 50% or greater decrease from baseline; symptom remission was defined as a score of 7 or lower. The GRID-HAMD assessment was audiorecorded to examine interrater reliability (eMethods in Supplement 2). Interrater reliability for all depression assessments (through postsession week 4) was 85%. Rapid and sustained antidepressant effects were examined at baseline; at day 1 and week 1 of postsession-1 follow-up; and at day 1, week 1, and week 4 postsession-2 follow-up using the Quick Inventory of Depressive Symptomatology–Self-Report (QIDS-SR; score range: 0-27, with higher scores indicating very severe depression).^39^

Descriptions of secondary outcome measures and timing of assessment are provided in the eMethods in Supplement 2. Secondary outcome measures for depressive symptoms were the Beck Depression Inventory II (score range: 0-63, with higher scores indicating severe depression)^40^ and the 9-item Patient Health Questionnaire (score range: 0-27, with higher scores indicating severe depression).^41^ The Columbia-Suicide Severity Rating Scale (severity of ideation subscale score range: 0-5, with higher scores indicating presence of ideation with at least some intent to die)^42,43^ was completed at every visit to assess for potentially worsening suicidal ideation throughout the trial. Anxiety symptoms were measured using the clinician-administered Hamilton Anxiety Rating Scale (score range: 0-56, with higher scores indicating severe anxiety)^44^ and the State-Trait Anxiety Index (score range: 0-80, with higher scores indicating greater anxiety).^45^ Blood pressure and heart rate were examined before and during the psilocybin sessions.

### Statistical Analysis

Data analysis was conducted on participants who completed the intervention (evaluable population). A previous study of psilocybin^16^ found a large effect of a high psilocybin dose (compared with a low dose) on reducing GRID-HAMD scores (Cohen *d* = 1.30). Assuming a similar large effect size with 24 participants, nearly 100% power was calculated to detect a statistically significant effect of psilocybin on change in depressive symptoms.

No primary outcome data were missing. Descriptive statistics for demographic and background characteristics for all study variables were calculated and compared between study conditions using a 2-sample *t* test for continuous variables and a χ^2^ test for all remaining variables. A repeated-measures analysis of variance with time (baseline, week 5, and week 8) and condition (immediate treatment and delayed treatment) as factors was used to examine changes in the primary depression outcome (GRID-HAMD score).

Follow-up planned comparisons included independent samples *t* tests to compare week 1 with week 4 GRID-HAMD scores in the immediate treatment condition group (corresponding to the week 5 and week 8 time points in the delayed treatment condition group). Within-participant (n = 24) treatment effect was examined using *t* tests comparing GRID-HAMD scores at baseline with scores at week 1 and week 4 postsession-2 follow-up. Rapid and sustained antidepressant effects were examined using *t* tests comparing QIDS-SR scores between baseline and day 1 postsession-1 and between baseline and week 4 postsession-2 follow-up. Effect sizes for the independent samples *t* tests were calculated using the Cohen *d* statistic, and effect sizes for the repeated-measures analysis of variance were calculated using the partial eta squared (η_p_^2^) statistic. Further primary outcomes included a descriptive analysis of the percentage of participants who met the criterion for clinically significant response and remission in the sample.

All statistical tests used a *P* < .05 to determine statistical significance. Data analysis was conducted from July 1, 2019, to July 31, 2020, using SPSS, version 25 (IBM).^46^ Data analysis plans for secondary outcomes are reported in the eMethods in Supplement 2.

## Results

A total of 27 participants were randomized, of whom 24 (89%) completed the intervention as well as the postsession assessments at weeks 1 and 4; specifically, 13 were randomized to the immediate treatment group and 11 to the delayed treatment group (Figure 1). The Table shows the demographic characteristics for the 24 participants, among whom were 16 women (67%) and 8 men (33%), with a mean (SD) age of 39.8 (12.2) years and a mean (SD) baseline GRID-HAMD score of 22.8 (3.9). An examination of the differences in stratification variables as a function of the treatment condition indicated no statistically significant differences between conditions (mean [SD] months in current major depressive episode: immediate treatment, 25.9 [22.4]; delayed treatment, 22.6 [22.5]; *P* = .39) (Table).

**Table.** Characteristics of the Overall Sample and Comparison of Baseline Demographic and Background Characteristics Between Participants in the Immediate and Delayed Treatment Condition Groups

Characteristic	No. (%)			χ^2^ or *t* Value^a^	*P* value^a^
	Overall sample (N = 24)	Immediate treatment (n = 13)	Delayed treatment (n = 11)		
Age, mean (SD), y	39.8 (12.2)	43.6 (13.0)	35.2 (9.9)	−1.8	.08
Time with depression, mean (SD), y	21.5 (12.2)	23.5 (12.7)	19.2 (11.8)	−0.86	.40
Time in current major depressive episode, mean (SD), mo^b^	24.4 (22.0)	25.9 (22.4)	22.6 (22.5)	−0.36	.39
Lifetime psychedelic use	0.8 (1.9)	0.5 (1.7)	1.3 (2.2)	1.02	.32
Female sex	16 (67)	9 (69)	7 (64)	1.34	.39
Heterosexual orientation	21 (96)	13 (100)	8 (89)	1.51	.41
White race/ethnicity	22 (92)	13 (100)	9 (82)	2.58	.20
Educational level					
<College	2 (8)	0 (0)	2 (18)	4.32	.41
Associate’s degree	2 (8)	1 (8)	1 (9)		
Bachelor’s degree	14 (58)	7 (54)	7 (64)		
Master’s degree	4 (17)	3 (23)	1 (9)		
Advanced degree	2 (8)	2 (15)	0 (0)		
Marital status					
Married/living with partner	11 (46)	6 (46)	5 (46)	0.94	>.99
Divorced/separated	1 (4)	1 (8)	0 (0)		
Never married	12 (50)	6 (46)	6 (55)		
Employment status					
Full-time	15 (63)	8 (62)	7 (64)	1.13	.73
Part-time	4 (17)	3 (23)	1 (9)		
Unemployed	5 (21)	2 (15)	3 (27)		

^a^ χ^2^, *t*, and *P* values refer to tests for differences between the immediate treatment and delayed treatment conditions.

^b^ Major depressive episode was defined by the *DSM-5*.

**Figure 3.**
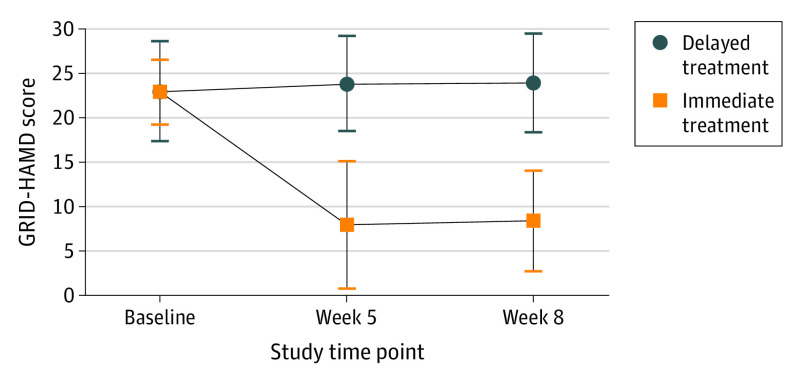
Comparison of GRID Hamilton Depression Rating Scale (GRID-HAMD) Scores Between the Delayed Treatment and Immediate Treatment Groups Data points are presented as mean (SD). In the immediate treatment group (n = 13), weeks 5 and 8 correspond to weeks 1 and 4 after the psilocybin session 2. In the delayed treatment group (n = 11), weeks 5 and 8 are prepsilocybin assessments obtained during the delay period. Effect sizes (Cohen *d* with 95% CI) and *P* values reflect the results of a 2-sample *t* test between the 2 groups at week 5 (Cohen *d* = 2.5; 95% CI, 1.4-3.5; *P* < .001) and week 8 (Cohen *d* = 2.6; 95% CI, 1.5-3.7; *P* < .001).

A statistically significant time by condition interaction effect on GRID-HAMD was found (η_p_^2^ = 0.57; 90% CI, 0.38-0.66; *P* < .001) (Figure 3).

Follow-up independent samples *t* tests revealed significantly lower depression scores in the immediate treatment condition at weeks 1 and 4 postsession-2 follow-up compared with the corresponding time points (weeks 5 and 8) in the delayed treatment condition before psilocybin treatment. In the immediate treatment group, the mean (SD) GRID-HAMD scores were 22.9 (3.6) at baseline, 8.0 (7.1) at week 5, and 8.5 (5.7) at week 8. In the delayed treatment group, the mean (SD) GRID-HAMD scores were 22.5 (4.4) at baseline, 23.8 (5.4) at week 5, and 23.5 (6.0) at week 8. The effect sizes were large at week 5 (Cohen *d* = 2.5; 95% CI, 1.4-3.5; *P* < .001) and at week 8 (Cohen *d* = 2.6; 95% CI, 1.5-3.7; *P* < .001) (eTables 1-3 and eResults in Supplement 2).

**Figure 4.**
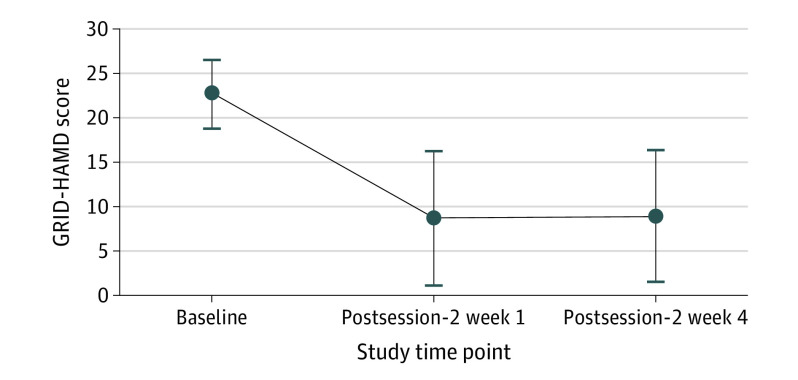
Decrease in the GRID Hamilton Depression Rating Scale (GRID-HAMD) Scores at Week 1 and Week 4 Postsession-2 Follow-up in the Overall Treatment Sample The mean (SD) GRID-HAMD score was 22.8 (3.9) at baseline, 8.7 (7.6) at week 1, and 8.9 (7.4) at week 4. Effect sizes (Cohen *d* with 95% CI) and *P* values reflect the results of a paired sample *t* test that compared scores between baseline and week 1 (Cohen *d* = 2.3; 95% CI, 1.5-3.1; *P* < .001) and week 4 postsession-2 follow-up (Cohen *d* = 2.3; 95% CI, 1.5-3.1; *P* < .001).

After the psilocybin session, 17 participants (71%) at week 1 and 17 participants (71%) at week 4 had a clinically significant response to the intervention (≥50% reduction in GRID-HAMD score), and 14 participants (58%) at week 1 and 13 participants (54%) at week 4 met the criteria for remission of depression (≤7 GRID-HAMD score). Within-participant *t* tests showed statistically significant decreases in GRID-HAMD scores among participants from baseline to week 1 (Cohen *d* = 2.3; 95% CI, 1.5-3.1; *P* < .001) and week 4 (Cohen *d* = 2.3; 95% CI, 1.5-3.1; *P* < .001) (Figure 4). The QIDS-SR measure of depression, which was assessed more frequently, showed a rapid, large decrease in mean (SD) depression score among participants from baseline to day 1 after psilocybin session 1 (16.7 [3.5] vs 6.3 [4.4]; Cohen *d* = 2.6; 95% CI, 1.8-3.5; *P* < .001). This substantial decrease remained through week 4 after session 2 (6.0 [5.7]; Cohen *d* = 2.3; 95% CI, 1.5-3.0; *P* < .001) (eFigure 1 in Supplement 2).

All secondary depression and anxiety outcomes showed a similar pattern of results as the primary depression outcomes, with statistically significant differences between conditions and across both conditions after entry into the active intervention period (eTables 1 to 3 and eFigures 1 to 8 in Supplement 2). For example, statistically significant treatment condition effects were found on self-reported depression (Beck Depression Inventory II and Patient Health Questionnaire–9) and clinician-administered anxiety (Hamilton Anxiety Rating Scale) measures. Overall, suicidal ideation was low and trended lower after enrollment in both groups (eFigure 9 in Supplement 2).

Participant and facilitator rated intensity of acute psilocybin effects are provided in eTables 4-6 in Supplement 2. There were no serious adverse events in this trial. A transient increase in blood pressure that exceeded the protocol criteria for more frequent assessment (ie, diastolic blood pressure >100 mm Hg) occurred during 1 session, but no medical intervention was needed, and the blood pressure level remained within predetermined safety parameters and resolved spontaneously during the session (eTable 7 in Supplement 2). Other nonserious adverse effects, which occurred during the psilocybin administration, that were reported by participants after completing at least one-half of the psilocybin sessions included challenging emotional (eg, fear and sadness) and physical (eg, feeling body shake or tremble) experiences (eTable 8 in Supplement 2). Mild to moderate transient headache was reported during 16 of 48 sessions (33%) and after the subjective psilocybin effects had subsided after 14 of 48 sessions (29%). Other adverse events are reported in eTables 8 and 9 in Supplement 2, and initiation of antidepressants or psychotherapy is reported in eTable 10 in Supplement 2.

## Discussion

This randomized clinical trial documented the substantial rapid and enduring antidepressant effects of psilocybin-assisted therapy among patients with MDD. Although the rapid antidepressant effects of psilocybin are similar to those reported with ketamine,^10,11^ the therapeutic effects are different: ketamine effects typically last for a few days to 2 weeks, whereas the current study showed that clinically significant antidepressant response to psilocybin therapy persisted for at least 4 weeks, with 71% of the participants continuing to show a clinically significant response (≥50% reduction in GRID-HAMD score) at week 4 of follow-up. Furthermore, psilocybin was found to have low potential for addiction^22^ and a minimal adverse event profile,^22,23^ suggesting therapeutic advantages with less risk for associated problems than ketamine.^12^ The present findings in patients with MDD are consistent with results of studies that reported on the effectiveness of psilocybin-assisted therapy in producing antidepressant effects among patients with cancer who had psychological distress^16,17,47^ and a small open-label study of patients with treatment-resistant depression.^18^

The mounting evidence of the use of psilocybin as an adjunct to treatment of a variety of psychiatric conditions (eg, depression,^16,17,18^ tobacco use disorder,^48^ and alcohol use disorder^49^) suggests a transdiagnostic mechanism of action. In several studies in patients^16,17,18,49,50,51^ and in healthy volunteers,^32,52^ the intensity of mystical-type experiences reported after psilocybin sessions was associated with favorable outcomes. Furthermore, cross-sectional studies have suggested that mystical-type and psychologically insightful experiences during a psychedelic session predict positive therapeutic effects.^53,54,55^ Consistent with these previous studies, the current trial showed that psilocybin-occasioned mystical-type, personally meaningful, and insightful experiences were associated with decreases in depression at 4 weeks (eResults in Supplement 2). Furthermore, a recent report suggested that psilocybin may decrease negative affect and the neural correlates of negative affect,^56^ which may be a mechanism underlying transdiagnostic efficacy. Taken together, these findings suggest that further studies into psychological and neural mechanisms across different psychiatric conditions are warranted.

The present trial showed that psilocybin administered in the context of supportive psychotherapy (approximately 11 hours) produced large, rapid, and sustained antidepressant effects. The effect sizes reported in this study were approximately 2.5 times greater than the effect sizes found in psychotherapy^57^ and more than 4 times greater than the effect sizes found in psychopharmacological depression treatment studies.^58^ These findings are consistent with literature that showed that combined pharmacotherapy and psychotherapy were more efficacious in the treatment of MDD than either intervention alone.^59,60,61^ Furthermore, given that psilocybin was associated with nonserious adverse effects that were frequently reported as mild-to-moderate headache and challenging emotions that were limited to the time of sessions (eTables 8 and 9 in Supplement 2), this intervention may be more acceptable to patients than widely prescribed antidepressant medications that confer substantially more problematic effects (eg, suicidal ideation, decrease in sexual drive, and weight gain). The effectiveness of psilocybin therapy after a single or only a few administrations represents another substantial advantage over commonly used antidepressants that require daily administration.

### Strengths and Limitations

This study has some strengths. It had a randomized design and used GRID-HAMD as the primary outcome measure that was assessed by blinded clinician raters. The delayed treatment condition controlled for the possible effects of having been accepted into the trial and for the passage of time between screening and initial follow-up assessments. However, the delayed treatment condition did not control for other aspects of psilocybin administration, such as preparation and rapport building, postsession integration meetings, or expectancy effects. Although placebo and active treatment controlled designs are widely used in therapeutic trials,^62^ they too have limitations owing to the highly discriminable effects of psilocybin.

This study has some other limitations. It had a short-term follow-up, a small sample that was predominantly composed of White non-Hispanic participants, and included participants with low risk of suicide and moderately severe depression. Further research with larger and more diverse samples, longer-term follow-up, and a placebo control is needed to better ascertain the safety (eg, abuse potential of psilocybin, suicide risk, and emergence of psychosis) and efficacy of this intervention among patients with MDD. Another limitation is the psychotherapy approach^31^ that involved session facilitators from a variety of professional disciplines (eg, social work, psychology, psychiatry) and session facilitators without formal clinical training (eg, research assistants and clinical trainees). The type of psychotherapy offered and the characteristics of therapists should be explored in future studies.

## Conclusions

Results of this randomized clinical trial demonstrated the efficacy of psilocybin-assisted therapy in producing large, rapid, and sustained antidepressant effects among patients with MDD. These data expand the findings of previous studies involving patients with cancer and depression as well as patients with treatment-resistant depression by suggesting that psilocybin may be effective in the much larger population of MDD. Further studies are needed with active treatment or placebo controls and in larger and more diverse populations.

## Notes

Supplement 1. Trial protocol

Supplement 2. eMethods eResults **eTable 1.** Repeated Measures ANOVAs Comparing Depression, Anxiety, and Suicidal Ideation Outcomes at Each Timepoint by Treatment Condition **eTable 2.** t Tests Comparing Depression, Anxiety, and Suicidal Ideation Outcomes at Each Timepoint by Condition **eTable 3.** Repeated Measures ANOVAs and Effect Sizes for Depression, Anxiety, and Suicidal Ideation Outcomes Across Overall Sample **eTable 4.** Ratings of Personal Meaning, Spiritual Significance, Psychological Challenge, and Psychological Insight During Each of Two Psilocybin Sessions **eTable 5.** Mean Proportion (and Standard Deviation) of Total Possible Score on the Mystical and Challenging Experiences Questionnaires During Each of Two Psilocybin Sessions; Proportion of Participants Who Had a Complete Mystical Experience in Each Session **eTable 6.** Monitor Ratings of Peak Psilocybin Effects During Each of Two Psilocybin Sessions **eTable 7.** Mean of the Peak Heart Rate and Blood Pressure Across Participants During Each of Two Psilocybin Sessions. Data Regarding Number of Participants Requiring Increased Rate of Monitoring **eTable 8.** Adverse Emotional and Physical Effects During Psilocybin Sessions **eTable 9.** Adverse Effects Reported the Day After Sessions 1 and 2 That Were Rated by Staff as Possibly or Probably Related to Psilocybin **eTable 10.** Initiation of Antidepressant Medication, Psychotherapy, or Psilocybin Reported 4 weeks After Session 2 **eFigure 1.** Decrease in Depression Scores on the Quick Inventory of Depression Symptoms (QIDS-SR) from Baseline to 1-day Post Psilocybin Session 1 and Through the 4-week Follow-up **eFigure 2.** Comparison of Depression Scores on the Quick Inventory of Depression Symptoms (QIDS-SR) by Treatment Condition **eFigure 3.** Comparison of Depression Scores on the Beck Depression Inventory – II (BDI-II) by Treatment Condition **eFigure 4.** Comparison of Depression Scores on the Patient Health Questionnaire – 9 Item (PHQ-9) by Treatment Condition **eFigure 5.** Comparison of Anxiety Scores on the Hamilton Anxiety Scale (HAM-A) by Treatment Condition (Immediate vs Delayed). Effect Size Calculation Using Cohen’s d Statistic **eFigure 6.** Comparison of Anxiety Scores on the State-Trait Anxiety Inventory – State Subscale (STAI-State) by Treatment Condition **eFigure 7.** Comparison of Anxiety Scores on the State-Trait Anxiety Inventory – Trait Subscale (STAI-Trait) by Treatment Condition **eFigure 8.** Comparison of Anxiety Scores on the State-Trait Anxiety Inventory – Total Scale (STAI-Total) by Treatment Condition **eFigure 9.** Comparison of Suicidal Ideation Scores on the Columbia Suicide Severity Rating Scale (CSSRS) by Treatment Condition

Supplement 3. Data sharing statement
