# Supplementary material for: Effects of Psilocybin-Assisted Therapy on Major Depressive Disorder: A Randomized Clinical Trial
Source: JAMA Psychiatry. 2020 Nov 4;78(5):1–9. doi: 10.1001/jamapsychiatry.2020.3285 (PMC7643046; doi:10.1001/jamapsychiatry.2020.3285)
Supplement: Supplement 1. — Trial protocol [file jamapsychiatry-e203285-s001.pdf]

Study Protocol
"Effects of psilocybin therapy for major depressive disorder: A randomized clinical trial"

8 July 2020

Alan K. Davis PhD[a,b], Frederick S. Barrett PhD[a], Darrick G. May MD[a], Mary P. Cosimano MSW[a], Nathan D. Sepeda BS[a], Matthew W. Johnson PhD[a], Patrick H. Finan PhD[a], Roland R. Griffiths PhD[a,c]

[a] Center for Psychedelic and Consciousness Research, Department of Psychiatry, Johns Hopkins School of Medicine, 5510 Nathan Shock Drive, Baltimore, MD 21224 USA

[b] College of Social Work, The Ohio State University, Columbus, OH 43210

[c] Department of Neuroscience, Johns Hopkins School of Medicine, 5510 Nathan Shock Drive, Baltimore, MD 21224 USA

Corresponding Authors:
Alan K. Davis, Ph.D. and Roland R. Griffiths, PhD., Center for Psychedelic and Consciousness Research, Department of Psychiatry, Johns Hopkins School of Medicine, 5510 Nathan Shock Drive, Baltimore, MD 21224 USA; Tele: 614-292-5251; Email: davis.5996@osu.edu and rgriff@jhmi.edu

Study Protocol

Study Overview: This study will involve a randomized waitlist control design to investigate the rapid and sustained antidepressant effects following two experimental sessions in which an oral dose of psilocybin is administered to patients with major depressive disorder. The study will include clinician and participant ratings of depression and anxiety pre- and post-drug-session, monitor and participant ratings of subjective drug effects during and after each drug session, tests of executive functioning at baseline and post-drug-session, and magnetic resonance imaging (MRI) of the brain at baseline and post-drug-session to assess the effects of psilocybin on cortical glutamate levels, neural response to emotional stimuli, and resting-state functional brain connectivity.

Twenty-four participants will complete two experimental drug sessions. Each session will last approximately eight hours, and experimental sessions will be separated by at least one week. Acute physiological, subjective, and behavioral effects will be assessed during and at the end of sessions (e.g., ratings completed by session monitors and questionnaire measures of subjective effects completed by participants), consistent with our well-established psilocybin session protocols. During the first experimental drug session, each participant will be administered a moderate dose of psilocybin (20 mg/70 kg), but one that we do not expect to have full therapeutic efficacy. The purpose of the first psilocybin session is to familiarize participants with the subjective effects that may be encountered at a higher dose. The first psilocybin session will also allow for assessment of both the suitability of each participant to the study procedures and drug effects, and the safety of administering a higher dose to each participant. The second session will occur about one week after the first session. If the study team decides that the participant can safely be administered the higher dose, then the participant will be given 30 mg/70 kg psilocybin during the second session. If the decision is made that the participant might clinically benefit from another session but not the high dose, the participant will receive 20 mg/70 kg psilocybin on the second session, with the dose determined by the clinical judgment of the study team.

Depressive symptoms will be assessed during visits at our research unit (the Behavioral Pharmacology Research Unit, or BPRU), at baseline and repeatedly after each session. Participants will also be contacted for clinical assessment 3, 6, and 12 months after the second experimental psilocybin session.

Waitlist Control Condition: After screening and enrollment, participants will be randomized to an immediate treatment group or a delayed treatment group (waitlist control condition). Participants in the delayed treatment group will wait 8 weeks after enrollment before beginning the study interventions and neuroimaging assessments. As a safety precaution, participants in the delayed treatment group will be assessed weekly via telephone calls or in-person visits during the wait period (i.e., telephone assessments during post-randomization weeks 1, 2, 3, 4, 6, and 7; in-person assessments during post-randomization weeks 5 and 8) to assess depressive symptoms and suicide risk to determine if intervention is warranted (as described in more detail in the section "Steps taken to minimize risk."

MRI Assessment: MRI measures will be collected for all 24 participants at two timepoints: 1. during the week before beginning the first treatment intervention; and 2. one week after the second psilocybin session. Only participants who successfully complete the initial MRI assessment will be eligible for inclusion in the post-treatment MRI assessment. [The MRI data are not reported in this primary study manuscript but will be reported later.]

We anticipate the following approximate timeline for study participation:

Screening and Preparation

Visits 1-2 (BPRU): Medical and psychological screening; cognitive testing; initial depressive symptom measures. Randomization to immediate or delayed treatment groups will occur at the end of Visit 2.

Telephone and in-person safety assessments for delayed treatment group: As a safety precaution, participants in the delayed treatment group will be assessed weekly via telephone calls or in-person visits during the 8-week wait period (i.e., telephone assessments during post-randomization weeks 1, 2, 3, 4, 6, and 7; in-person assessments during post-randomization weeks 5 and 8) to assess depressive symptoms and suicide risk. For clarity in describing the study procedures, none of these safety assessments are designated as a study "visit." In addition to the safety assessments at weeks 5 and 8, primary outcome assessments (GRID-HAMD) will also be conducted.

Visit 3: Immediate treatment group: Initial MRI assessment and neurocognitive testing (KKI; 1 hour). This visit will occur within about one week after Visit 2.

Delayed treatment group: Initial MRI assessment and neurocognitive testing (KKI; 1 hour). This visit will occur about 8 weeks after Visit 2. In addition to the brain imaging and neurocognitive testing, depressive symptom measures will be reassessed in the delayed treatment group (BPRU; 1 hour).

Visits 4-5: Preparation for the first psilocybin session consists of 2 meetings over 2 weeks (BPRU; 6 hours total). Visit 4 will occur within about 1 week after Visit 3. Visit 5 will occur about one week after Visit 4.

Psilocybin Session 1 and follow-up visits

Visit 6: First psilocybin session (20 mg/70 kg) at BPRU; clinical, physiological, & behavioral measures (8 hours total). Visit 6 will occur within about 1 week of Visit 5.

Visit 7: Post-session 1 integration; meeting with study clinician & staff; clinical & behavioral measures (BPRU); (2 hours total). Visit 7 will occur within 3 days after Visit 6.

Visit 8: 1-week post-session 1; meeting with study clinician & staff; clinical & behavioral measures (BPRU) (2 hours total)

Psilocybin Session 2 and follow-up visits

Visit 9: Second psilocybin session (20 or 30 mg/70 kg) at BPRU; clinical, physiological, & behavioral measures (8 hours total). Visit 9 will occur within about 1 week of Visit 8.

Visit 10: Post-session 2 integration; meeting with study clinician & staff; clinical & behavioral measures (BPRU); (2 hours total). Visit 10 will occur within 3 days after Visit 9.

Visit 11: Week 1 post-session 2; meeting with study clinician & staff; clinical & behavioral measures; cognitive testing (BPRU; 3 hours total); brain imaging for all participants (KKI) (1 hour total)

Visit 12: Week 2 post-session 2 – clinical & behavioral ratings

Telephone safety assessment: Week 3 post-session 2 – To assure at least weekly safety assessment for depressive symptoms and suicide risk during the intervention period (for both treatment groups), there will be one telephone safety assessment (in between Visits 12 and 13). This telephone safety assessment is not designated as a study "visit."

Visit 13: Week 4 post-session 2 – meeting with study clinician & staff; clinical & behavioral measures; cognitive testing (BPRU); (3 hours total)

Additional visits for immediate treatment group only

Visit 14 & 15: Week 9 & Week 12 post-session 2 – Participants in immediate treatment group will come to BPRU for assessment of depressive symptoms (1 hour). The purpose of these visits is to keep the number and timepoints of primary outcome assessments consistent between treatment groups.

Long-term follow-up (LTFU)

LTFU 1: 3 months post-session 2; questionnaire assessments (2 hours total)

LTFU 2: 6-months post-session 2; open-ended clinical interview; questionnaire assessments (2 hours total)

LTFU 3: 12-months post-session 2; questionnaire assessments (2 hours total)

Preparation Meetings with Monitors Before and After Session Days: Before the first session, participants will meet with one or more of the session monitors on at least 2 occasions, for a total of about 6 hours of contact time before the first session day. The main purpose of the participant-monitor meetings is to develop rapport and trust, which we believe helps minimize the risk of fear or anxiety reactions during the psilocybin sessions. Additional meetings and contact hours will be scheduled if it is judged necessary to establish rapport and trust. Consistent with our previous psilocybin protocols, the participant's life history and current situation in life will be reviewed, and intentions and expectations for the psilocybin sessions will be discussed. Details regarding the conduct of the supportive psychotherapy provided have been published elsewhere.[1]

The participant will meet with the session monitor(s) one day and one week after each session and four weeks after the second session to support integration of session-day experiences. Additional clinician and monitor contact hours will be scheduled if it is judged that the participant would benefit from additional meetings in order to discuss experiences from their session(s) or prepare for the next session.

Session Monitor Telephone Meetings: In order to provide ongoing clinical support after the session experiences, telephone discussions may occur throughout study participation if the clinician, or monitor judge that this additional support would be helpful.

Conduct of Psilocybin Sessions (Visits 6 and 9): Procedures for psilocybin administration and the conduct of the session will be similar to procedures used in our previous[2,3] and ongoing studies with psilocybin. Participants will be instructed to consume a low-fat breakfast before reporting to the laboratory for the psilocybin sessions. Before psilocybin administration, the participant will provide a urine sample, which will be tested for the presence of abused drugs (e.g., various opioids, stimulants and sedatives). In addition, a urine pregnancy test will be conducted in females of child bearing potential. A negative pregnancy test will be required in order to continue in the study. Participants and staff will also complete pre-session questionnaires. Before each psilocybin session, study personnel will interview the participant. If

the investigator believes that the session is contraindicated, the session will be cancelled or postponed.

Psilocybin will be administered in opaque gelatin capsules with approximately 100 ml water. At least one session monitor, under the supervision of the investigators, will be present in the room and available to respond to participants' physical and emotional needs during the full course of the session (at least 7 hours). A physician on the study team will be immediately available via pager or mobile phone for at least 3 hours or until the peak effects of psilocybin have subsided, whichever is longer.

During the session, participants will lie on a couch, wear eyeshades, and listen to a program of music through headphones. The participant will be encouraged to focus her or his attention inward. The eyeshades and music are intended to encourage this inward reflection.

Heart rate and blood pressure will be measured pre drug administration, at 30, 60, 90, 120 minutes after drug administration, and then at least hourly until at least 6 hours after drug administration and until drug effects have subsided. Heart rate and blood pressure assessments will be obtained after the participant has been sitting or recumbent for at least 5 minutes. Sessions are expected to last approximately 7 to 10 hours.

At about the same time of each heart rate/blood pressure measurement, monitors will complete questionnaires to rate the presence and intensity of behaviors, signs, and reported symptoms, including sleepiness, amount of speech, anxiety, stimulation/arousal, tearing/crying, nausea/vomiting, yawning, restlessness, feelings of unreality, visual changes, euphoria, and peacefulness. Video and audio recordings will be made throughout the session.

At the end of the experimental session, participants will complete paper or computer-based questionnaires designed to assess acute subjective experiences associated with the psilocybin session. Study monitors will complete assessments of mood and safety. Subjects will also be asked to write a narrative description of the experience of the psilocybin session before their next in-person meeting.

<u>In-person post-session meetings (Visits 7, 8, 10, and 11)</u>: During the meetings with the monitors after each session (1-3 days post-session, or Visits 7 and 10, and 1 week post-session, or Visits 8 and 11), participants will complete questionnaire assessment measures. During visits 7 and 10, the monitors will also complete questionnaires retrospectively rating various behaviors and experiences observed on the session day and reported by the participant during the meeting (e.g., Next-Day Monitor Rating Form), and participants will be asked to recite and discuss the narrative description of their most recent psilocybin session.

<u>Long-term follow-up visits</u>: [Long-term follow-up assessment is ongoing and results will be reported later.]

<u>Timing and Location of Meetings, Sessions, and Measures</u>: Although much effort is put in to scheduling volunteers within the stated time frames, these are only estimates. Variables out of the study team's control (e.g., volunteer availability, university closings, availability of study rooms, illness) may prevent the scheduling of volunteers within the stated time frame goals. There is no evidence to suggest that the difference in timing of sessions, meetings, and measures will adversely affect the volunteer or the validity of the study. On those occasions when the timing of meetings, sessions or measures deviate substantially from the time frames in the protocol, we will report these as deviations in the Continuing Review.

Inclusion/Exclusion Criteria

Overview of Screening Procedures: Participants will be initially screened via telephone or an online questionnaire to determine whether they meet major inclusion/exclusion criteria, and thus whether they are eligible for an in-person screening session.

Participants who do not fail telephone or online screening will be invited to BPRU on the Johns Hopkins Bayview Campus for in-person screening.

Written informed consent will be obtained at the BPRU at a scheduled meeting after participants have passed telephone screening. Participants will be allowed to take as much time as necessary to decide whether or not to sign the consent form. Study staff will discuss the consent form with the participant after the participant has read the consent form. Study staff will ask questions to assess the individual's understanding of the consent form. Participants may take the consent form home to review and return to sign consent if they wish.

Participants will be physically healthy adult participants (approximately equal numbers of males and females) 21 to 75 years old who have a DSM-5 diagnosis of major depressive disorder, and are currently experiencing a major depressive episode. Potential participants will self-report their history of antidepressant use and the effectiveness of antidepressant use during the initial telephone or online screening. Persons currently taking an antidepressant will be excluded at this initial screening. Potential participants that have previously used antidepressant medications will provide a written list of these medications.

Non-English speakers and those with language or hearing impairments will not participate in the study. Participants will be recruited primarily from the Baltimore/Washington DC area. Participants may or may not have used hallucinogens in the past. Potential participants will be carefully screened to eliminate those with significant medical or psychiatric illnesses other than major depressive disorder (see below for specific inclusion/exclusion criteria).

Screening evaluation will include a history and physical examination, ECG, a 30 cc blood draw for study measures and medical screening, a personal and family medical history questionnaire, psychiatric/psychological assessments, and a urine drug test. As per standard BPRU screening procedures, screening physical examination and ECGs will be performed by BPRU medical staff (nurse, mid-level, or physician). ECGs will be interpreted by cardiologists credentialed and privileged to read ECGs at Bayview Medical Center to determine if a significant abnormality exists. Participants will be requested to refrain from illicit drug use during the course of the study and a urine test will be conducted before each drug session (e.g., testing for various opioids, stimulants and sedatives). Pregnant or nursing women are ineligible; female participants will receive a urine pregnancy test at intake and before each drug session, and must use effective methods of contraception during the study. Psychiatric screening will be conducted by study team members. Psychoactive drug-use history, history of antidepressant treatments, and information about employment status and current functioning (including mood and psychological and psychosomatic symptoms) will be obtained. All inclusion and exclusion criteria are described below.

Based on testing and interviews, if a psychiatric/psychological problem of a crisis nature (e.g., acute psychosis or suicidal intent) is discovered during screening, we will refer that individual to the Emergency Department for treatment.

Study participants will not be enrolled if they are currently taking antidepressant medication. Study physicians will assume the role of mental health provider upon participants enrolling in the study through the final primary outcome timepoint (Visit 13, 4 weeks post-psilocybin session 2).

<u>Inclusion criteria</u>:

- 21 to 75 years old
- Have given written informed consent
- Have at least a high-school level of education or equivalent (e.g. GED).
- Have a confirmed DSM-5 diagnosis of Major Depressive Disorder and currently experiencing a major depressive episode.
- Have a baseline GRID-HAMD score greater than or equal to 17.
- No antidepressant medication for at least 2 weeks (4 weeks for fluoxetine) prior to enrollment.
- Be judged by study team clinicians to be at low risk for suicidality
- Concurrent psychotherapy is allowed if the type and frequency of the therapy has been stable for at least two months prior to screening and is expected to remain stable during participation in the study.
- Be medically stable as determined by screening for medical problems via a personal interview, a medical questionnaire, a physical examination, an electrocardiogram (ECG), and routine medical blood and urinalysis laboratory tests
- Agree to consume approximately the same amount of caffeine-containing beverage (e.g., coffee, tea) that he/she consumes on a usual morning, before arriving at the research unit on the mornings of drug session days. If the participant does not routinely consume caffeinated beverages, he/she must agree not to do so on session days.
- Agree to refrain from using any psychoactive drugs, including alcoholic beverages and nicotine, within 24 hours of each drug administration. The exception is caffeine. Participants will be required to be non-smokers.
- Agree not to take any PRN medications on the mornings of drug sessions
- Agree not to take sildenafil (Viagra®), tadalafil, or similar medications within 72 hours of each drug administration.
- Agree that for one week before each drug session, he/she will refrain from taking any nonprescription medication, nutritional supplement, or herbal supplement except when approved by the study investigators. Exceptions will be evaluated by the study investigators and will include acetaminophen, non-steroidal anti-inflammatory drugs, and common doses of vitamins and minerals.
- Have limited lifetime use of hallucinogens (the following criteria are preferred: no use in the past 5 years; total hallucinogen use less than 10 times)

<u>General medical exclusion criteria</u>:

- Women who are pregnant (as indicated by a positive urine pregnancy test assessed at intake and before each drug session) or nursing; women who are of child-bearing potential and sexually active who are not practicing an effective means of birth control.
- Cardiovascular conditions: coronary artery disease, stroke, angina, uncontrolled hypertension, a clinically significant ECG abnormality (e.g., atrial fibrilation), prolonged QTc interval (i.e., QTc > 450 msec), artificial heart valve, or TIA in the past year
- Epilepsy with history of seizures

- Insulin-dependent diabetes; if taking oral hypoglycemic agent, then no history of hypoglycemia
- Currently taking psychoactive prescription medication on a regular (e.g., daily) basis
- Currently taking on a regular (e.g., daily) basis any medications having a primary centrally-acting serotonergic effect, including MAOIs. For individuals who have intermittent or PRN use of such medications, psilocybin sessions will not be conducted until at least 5 half-lives of the agent have elapsed after the last dose.
- More than 25% outside the upper or lower range of ideal body weight according to Metropolitan Life height and weight table

Psychiatric Exclusion Criteria:
- Current or past history of meeting DSM-5 criteria for schizophrenia spectrum or other psychotic disorders (except substance/medication-induced or due to another medical condition), or Bipolar I or II Disorder
- Current or history within one year of meeting DSM-5 criteria for a moderate or severe alcohol, tobacco, or other drug use disorder (excluding caffeine)
- Have a first or second-degree relative with schizophrenia spectrum or other psychotic disorders (except substance/medication-induced or due to another medical condition), or Bipolar I or II Disorder
- Has a psychiatric condition judged to be incompatible with establishment of rapport or safe exposure to psilocybin
- History of a medically significant suicide attempt
- Has failed to respond to electroconvulsive therapy during the current major depressive episode
- Current antidepressant use

Additional MRI Exclusion Criteria:
- Head trauma
- Claustrophobia incompatible with scanning
- Cardiac pacemaker
- Implanted cardiac defibrillator
- Aneurysm brain clip
- Inner ear implant
- Prior history as a metal worker and/or certain metallic objects in the body -- must complete MRI screening form (see eIRB Study Documents) and be approved by MRI technologist before each scan
- History of clinically significant vertigo, seizure disorder, middle ear disorder, or double vision
- Poor vision not adequately corrected (in order to complete emotional processing task)

Drugs/ Substances/ Devices

Psilocybin dose: All participants will receive a moderate dose of 20 mg/70 kg on the first psilocybin session. The psilocybin dose for the second session will be increased to 30 mg/70 kg unless a participant experiences a strong unpleasant reaction during the first session and the clinical judgment of the study team is that the given participant would have a similar or greater reaction at a higher dose that would reduce the possible therapeutic benefits of psilocybin.

Primary outcome variable.

Depression Measures: The primary outcome measure for depressive symptoms is the 17-item GRID-Hamilton Depression Rating Scale (GRID-HAMD)[4,5] assessed by blinded clinician raters. All participants will have this measured at screening (Visit 2), at post-randomization weeks 5, 8, 13, and 16, and at long-term follow-ups. Post-randomization weeks 5 and 8 correspond to the primary endpoints of 1 and 4 weeks post-psilocybin session 2 for the immediate treatment group; weeks 13 and 16 correspond to primary endpoints for the delayed treatment group. Having the same number and timepoints of primary outcome assessments will allow for between-group comparisons. This scale assesses severity of depressive symptoms with a higher score indicating more severe depression. Screening measurements for the immediate treatment group will be considered as baseline measurements for this group, and measurements at the end of the wait period for the delayed treatment group will be considered baseline measurements for this group for the purposes of assessing treatment outcomes.

Secondary outcome variables.

Depression Measures: Secondary outcome measures for depressive symptoms are the Beck Depression Inventory II (BDI-II),[6] the 9-item Patient Health Questionnaire (PHQ-9),[7] and the Quick Inventory of Depressive Symptomatology – Self Rated (QIDS-SR).[8,9] These will be administered at the same timepoints as the primary depression measures.

Safety Measures: The Columbia Suicide Severity Rating Scale (C-SSRS)[10] will be used to assess severity of suicide ideation during every in-person visit. It was developed by researchers at Columbia University and is widely used in clinical and research settings (www.cssrs.columbia.edu). As a safety precaution, participants in the delayed treatment group will have weekly assessments of depressive symptoms (using the PHQ-9) and suicide risk (using the C-SSRS) to determine if intervention is warranted.

Measures taken throughout each drug session:

Heart rate and blood pressure: Vital signs will be assessed before capsule administration and at 30, 60, 90, 120, 180, 240, 300, and 360 minutes after capsule administration.

Within-session Monitor Rating Form: At the same time points at which the heart rate and blood pressure will be taken, the two session monitors will complete the Monitor Rating Form, which involves rating or scoring several dimensions of the participant’s behavior and mood.

Video and audio recordings: Video and audio recordings may be taken throughout the drug sessions and will be reviewed by investigators.

Measures taken about 7 hours after capsule administration:

Mystical Experience Questionnaire (MEQ30)[11,12]

Challenging Experience Questionnaire (CEQ27)[13]

Longitudinal measures assessed at baseline, post-session time-points, and follow-up:

Anxiety and emotion regulation rating scales: Participants will complete a number of questionnaires that assess anxiety (State Trait Anxiety Inventory, or STAI,[14] and Hamilton Anxiety Inventory, or HAM-A)[15] during screening and 1 week and 4 weeks after Session 2.

Measures taken 4 weeks, 3 months, 6 months, and 12 months after Session 2:

Persisting Effects Questionnaire: This questionnaire was developed to assess attributions that participants retrospectively make with respect to the psilocybin experience.[2,3,16]

Monitor Rating of Enduring Effects: This questionnaire will assess the monitors' observations of persisting effects of sessions on participant attitudes and behaviors.

Measures taken at baseline only:

Antidepressant Treatment Response Questionnaire (ATRQ): If a participant has a history of antidepressant use, we will use the ATRQ to determine effectiveness of past trials for purposes of characterizing that participant's degree of treatment-resistance.[17–20]

[Other tertiary measures were examined in this trial that are not reported here. These measures will be included in forthcoming manuscripts.]

Statistical plan.

Questionnaire Measures: Repeated-measures ANOVA will be used when appropriate to assess change between groups and over time within groups in primary and secondary longitudinal questionnaire outcome measures, including measures of depressive symptoms and executive function. Planned comparisons will be used to test for early antidepressant effects (between baseline and 1-week post-session ratings), and to test for sustained antidepressant effects (between baseline and 4-week post-session ratings) of psilocybin. Planned comparisons will also be used to investigate change in questionnaire and executive function test scores between pre-session (e.g. Visits 2, 5 and 8) and post-session (e.g. Visits 7, and 10 - 15) timepoints for secondary outcome measures, and between depressive assessments of immediate and delayed treatment groups at 5 and 8 weeks post-randomization. We will also assess correlations between primary and secondary outcome measures.

References

1. Johnson MW, Richards WA, Griffiths RR. Human hallucinogen research: guidelines for safety. *J Psychopharmacol (Oxf)*. 2008;22(6):603-620. doi:10.1177/0269881108093587

2. Griffiths RR, Richards WA, McCann U, Jesse R. Psilocybin can occasion mystical-type experiences having substantial and sustained personal meaning and spiritual significance. *Psychopharmacology (Berl)*. 2006;187(3):268-283. doi:10.1007/s00213-006-0457-5

3. Griffiths RR, Johnson MW, Richards WA, Richards BD, McCann U, Jesse R. Psilocybin occasioned mystical-type experiences: immediate and persisting dose-related effects. *Psychopharmacology (Berl)*. 2011;218(4):649-665. doi:10.1007/s00213-011-2358-5

4. Hamilton M. A rating scale for depression. *J Neurol Neurosurg Psychiatry*. 1960;23(1):56-62. doi:10.1136/jnnp.23.1.56

5. Williams JBW, Kobak KA, Bech P, et al. The GRID-HAMD: standardization of the Hamilton Depression Rating Scale. *Int Clin Psychopharmacol*. 2008;23(3):120-129. doi:10.1097/YIC.0b013e3282f948f5

6. Beck AT, Steer RA, Ball R, Ranieri WF. Comparison of Beck Depression Inventories-IA and-II in psychiatric outpatients. *J Pers Assess*. 1996;67(3):588-597. doi:10.1207/s15327752jpa6703_13

7. Kroenke K, Spitzer RL. The PHQ-9: a new depression diagnostic and severity measure. *Psychiatr Ann*. 2002;32(9):509-515. doi:10.3928/0048-5713-20020901-06

8. Lenderking WR, Hu M, Tennen H, Cappelleri JC, Petrie CD, Rush AJ. Daily process methodology for measuring earlier antidepressant response. *Contemp Clin Trials*. 2008;29(6):867-877. doi:10.1016/j.cct.2008.05.012

9. Rush AJ, Trivedi MH, Ibrahim HM, et al. The 16-Item quick inventory of depressive symptomatology (QIDS), clinician rating (QIDS-C), and self-report (QIDS-SR): a psychometric evaluation in patients with chronic major depression. *Biol Psychiatry*. 2003;54(5):573-583. doi:10.1016/S0006-3223(02)01866-8

10. Posner K, Brent D, Lucas C, et al. *Columbia-Suicide Severity Rating Scale (C-SSRS)*. Columbia University Medical Center; 2008.

11. MacLean KA, Johnson MW, Griffiths RR. Mystical experiences occasioned by the hallucinogen psilocybin lead to increases in the personality domain of openness. *J Psychopharmacol (Oxf)*. 2011;25(11):1453-1461. doi:10.1177/0269881111420188

12. Barrett FS, Johnson MW, Griffiths RR. Validation of the revised Mystical Experience Questionnaire in experimental sessions with psilocybin. *J Psychopharmacol (Oxf)*. 2015;29(11):1182-1190. doi:10.1177/0269881115609019

13. Barrett FS, Bradstreet MP, Leoutsakos J-MS, Johnson MW, Griffiths RR. The Challenging Experience Questionnaire: Characterization of challenging experiences with psilocybin

mushrooms. *J Psychopharmacol (Oxf)*. 2016;30(12):1279-1295. doi:10.1177/0269881116678781

14. Spielberger CD, Sydeman SJ. State-Trait Anxiety Inventory and State-Trait Anger Expression Inventory. In: *The Use of Psychological Testing for Treatment Planning and Outcome Assessment*. Lawrence Erlbaum Associates, Inc; 1994:292-321.

15. Hamilton M. The assessment of anxiety states by rating. *Br J Med Psychol*. 1959;32(1):50-55. doi:10.1111/j.2044-8341.1959.tb00467.x

16. Griffiths R, Richards W, Johnson M, McCann U, Jesse R. Mystical-type experiences occasioned by psilocybin mediate the attribution of personal meaning and spiritual significance 14 months later. *J Psychopharmacol (Oxf)*. 2008;22(6):621-632. doi:10.1177/0269881108094300

17. Chandler GM, Iosifescu DV, Pollack MH, Targum SD, Fava M. Research: validation of the Massachusetts General Hospital Antidepressant Treatment History Questionnaire (ATRQ): Validation of the MGH ATRQ. *CNS Neurosci Ther*. 2010;16(5):322-325. doi:10.1111/j.1755-5949.2009.00102.x

18. Thase ME, Youakim JM, Skuban A, et al. Efficacy and Safety of Adjunctive Brexpiprazole 2 mg in Major Depressive Disorder: A Phase 3, Randomized, Placebo-Controlled Study in Patients With Inadequate Response to Antidepressants. *J Clin Psychiatry*. 2015;76(09):1224-1231. doi:10.4088/JCP.14m09688

19. Ball SG, Ferguson MB, Martinez JM, et al. Efficacy Outcomes From 3 Clinical Trials of Edivoxetine as Adjunctive Treatment for Patients With Major Depressive Disorder Who Are Partial Responders to Selective Serotonin Reuptake Inhibitor Treatment. *J Clin Psychiatry*. 2016;77(05):635-642. doi:10.4088/JCP.14m09619

20. Ionescu DF, Swee MB, Pavone KJ, et al. Rapid and Sustained Reductions in Current Suicidal Ideation Following Repeated Doses of Intravenous Ketamine: Secondary Analysis of an Open-Label Study. *J Clin Psychiatry*. 2016;77(06):e719-e725. doi:10.4088/JCP.15m10056
