# Supplementary material for: Effects of Psilocybin-Assisted Therapy on Major Depressive Disorder: A Randomized Clinical Trial
Source: JAMA Psychiatry. 2020 Nov 4;78(5):1–9. doi: 10.1001/jamapsychiatry.2020.3285 (PMC7643046; doi:10.1001/jamapsychiatry.2020.3285)
Supplement: Supplement 2. — eMethods eResults eTable 1. Repeated Measures ANOVAs Comparing Depression, Anxiety, and Suicidal Ideation Outcomes at Each Timepoint by Treatment Condition eTable 2. t Tests Comparing Depression, Anxiety, and Suicidal Ideation Outcomes at Each Timepoint by Condition eTable 3. Repeated Measures ANOVAs and Effect Sizes for Depression, Anxiety, and Suicidal Ideation Outcomes Across Overall Sample eTable 4. Ratings of Personal Meaning, Spiritual Significance, Psychological Challenge, and Psychological Insight During Each of Two Psilocybin Sessions eTable 5. Mean Proportion (and Standard Deviation) of Total Possible Score on the Mystical and Challenging Experiences Questionnaires During Each of Two Psilocybin Sessions; Proportion of Participants Who Had a Complete Mystical Experience in Each Session eTable 6. Monitor Ratings of Peak Psilocybin Effects During Each of Two Psilocybin Sessions eTable 7. Mean of the Peak Heart Rate and Blood Pressure Across Participants During Each of Two Psilocybin Sessions. Data Regarding Number of Participants Requiring Increased Rate of Monitoring eTable 8. Adverse Emotional and Physical Effects During Psilocybin Sessions eTable 9. Adverse Effects Reported the Day After Sessions 1 and 2 That Were Rated by Staff as Possibly or Probably Related to Psilocybin eTable 10. Initiation of Antidepressant Medication, Psychotherapy, or Psilocybin Reported 4 weeks After Session 2 eFigure 1. Decrease in Depression Scores on the Quick Inventory of Depression Symptoms (QIDS-SR) from Baseline to 1-day Post Psilocybin Session 1 and Through the 4-week Follow-up eFigure 2. Comparison of Depression Scores on the Quick Inventory of Depression Symptoms (QIDS-SR) by Treatment Condition eFigure 3. Comparison of Depression Scores on the Beck Depression Inventory – II (BDI-II) by Treatment Condition eFigure 4. Comparison of Depression Scores on the Patient Health Questionnaire – 9 Item (PHQ-9) by Treatment Condition eFigure 5. Comparison of Anxiety Scores on the H [file jamapsychiatry-e203285-s002.pdf]

Supplementary Online Content

Davis AK, Barrett FS, May DG, et al. Effects of psilocybin-assisted therapy on major depressive disorder: a randomized clinical trial. *JAMA Psychiatry*. Published online November 4, 2020. doi:10.1001/jamapsychiatry.2020.3285

eMethods

eResults

eTable 1. Repeated Measures ANOVAs Comparing Depression, Anxiety, and Suicidal Ideation Outcomes at Each Timepoint by Treatment Condition

eTable 2. t Tests Comparing Depression, Anxiety, and Suicidal Ideation Outcomes at Each Timepoint by Condition

eTable 3. Repeated Measures ANOVAs and Effect Sizes for Depression, Anxiety, and Suicidal Ideation Outcomes Across Overall Sample

eTable 4. Ratings of Personal Meaning, Spiritual Significance, Psychological Challenge, and Psychological Insight During Each of Two Psilocybin Sessions

eTable 5. Mean Proportion (and Standard Deviation) of Total Possible Score on the Mystical and Challenging Experiences Questionnaires During Each of Two Psilocybin Sessions; Proportion of Participants Who Had a Complete Mystical Experience in Each Session

eTable 6. Monitor Ratings of Peak Psilocybin Effects During Each of Two Psilocybin Sessions

eTable 7. Mean of the Peak Heart Rate and Blood Pressure Across Participants During Each of Two Psilocybin Sessions. Data Regarding Number of Participants Requiring Increased Rate of Monitoring

eTable 8. Adverse Emotional and Physical Effects During Psilocybin Sessions

eTable 9. Adverse Effects Reported the Day After Sessions 1 and 2 That Were Rated by Staff as Possibly or Probably Related to Psilocybin

eTable 10. Initiation of Antidepressant Medication, Psychotherapy, or Psilocybin Reported 4 weeks After Session 2

eFigure 1. Decrease in Depression Scores on the Quick Inventory of Depression Symptoms (QIDS-SR) from Baseline to 1-day Post Psilocybin Session 1 and Through the 4-week Follow-up

eFigure 2. Comparison of Depression Scores on the Quick Inventory of Depression Symptoms (QIDS-SR) by Treatment Condition
eFigure 3. Comparison of Depression Scores on the Beck Depression Inventory – II (BDI-II) by Treatment Condition

eFigure 4. Comparison of Depression Scores on the Patient Health Questionnaire – 9 Item (PHQ-9) by Treatment Condition

eFigure 5. Comparison of Anxiety Scores on the Hamilton Anxiety Scale (HAM-A) by Treatment Condition (Immediate vs Delayed). Effect Size Calculation Using Cohen's d Statistic

eFigure 6. Comparison of Anxiety Scores on the State-Trait Anxiety Inventory – State Subscale (STAI-State) by Treatment Condition

eFigure 7. Comparison of Anxiety Scores on the State-Trait Anxiety Inventory – Trait Subscale (STAI-Trait) by Treatment Condition

eFigure 8. Comparison of Anxiety Scores on the State-Trait Anxiety Inventory – Total Scale (STAI-Total) by Treatment Condition

eFigure 9. Comparison of Suicidal Ideation Scores on the Columbia Suicide Severity Rating Scale (CSSRS) by Treatment Condition

This supplementary material has been provided by the authors to give readers additional information about their work.

©2020 Davis AK et al. *JAMA Psychiatry*.

eMethods

Music Playlist

PLAYLIST, MAJOR DEPRESSIVE DISORDER STUDY
JOHNS HOPKINS SCHOOL OF MEDICINE

Arrival and Ingestion*

MINUTES	SECONDS	
3	32	Antonio Vivaldi, Andante, Concerto RV 532 in G Major for 2 guitars, strings & continuo, Guitar Concertos, Los Romeros, Academy of St. Martin-in-the-Fields, Philips 412-624-2.
3	55	Antonio Vivaldi, Largo, Concerto RV93 in D Major for guitar, strings & continuo, Ibid.
2	22	Antonio Vivaldi, Largo, Concerto RV 356 in A Minor for guitar, Ibid.
3	26	Paul Horn "Mumtaz Mahal", Inside the Taj Mahal, Kuckuck, LC2099.
5	42	Paul Horn "Shah Jahan", Inside the Taj Mahal, Ibid.
2	22	Ron Korb, "Flute Traveller (Alto Flute), Oasis Productions Limited, SOCAN NHCD 205.
6	46	JS Bach: Suite No. 3 (Bach) Brazilian Guitar Quartet, Delos B00004YR6P
10	57	Russill Paul "By the Stream" (PM Yoga Chants), Gaiam, The Relaxation Company, CD 3142.
2	32	"Om Namah Shivaya" (CD from "Yoga of Sound"), Novato CA: New World Library, 2004.
6	18	Edward Elgar, "Nimrod", Enigma Variation #9, Leonard Bernstein, The Artist's Album, DGG 457 691-2.
6	17	Morten Lauridsen "O Magnum Mysterium", A Robert Shaw Christmas: Angels on High, Telarc20 CD-80461.
5	32	"Alleluia, Behold the Bridegroom", Sacred Treasures III: Choral Masterworks from Russia and Beyond, St. Petersburg Chamber Choir, Hearts of Space, 025041111423.

59 minutes, 41 seconds

26	49	Henryk Gorecki, "Lento--Sostenuto Tranquillo ma Cantabile" (Symphony #3, Movement #1), London Sinfonietta, David Zinman, Dawn Upshaw, Elektra Nonesuch 9 79282-2.
10	41	Johannes Brahms, "Selig sind, die da Leid tragen" (Ein Deutches Requiem), San Francisco Symphony & Chorus, Herbert Blomstedt, London 443 771-2.
14	39	Johannes Brahms, "Denn alles Fleish es ist wie Gras" (Ein Deutsches Requiem), Ibid.

©2020 Davis AK et al. *JAMA Psychiatry*.

10	16	Johannes Brahms, "Adagio, Non Troppo" (Symphony No. 2, Op. 73), New York Philharmonic, Leonard Bernstein, Sony SMK 61829.
5	42	Johannes Brahms, "Wie lieblich sind Deine Wohnungen" (Ein Deutsches Requiem), Op. cit.
10	26	J.S. Bach, "Kyrie" (Mass in B Minor), Robert Shaw, Atlanta Symphony & Chamber Chorus, Telarc CDE-80233.
4	28	J.S. Bach, Completion of "Kyrie" (Omiting "Christe Eleison" duet)

1 hour, 23 minutes, 01 second

10	2	Samuel Barber, "Adagio for Strings" New York Philharmonic, Leonard Bernstein, Sony, SMK 63088
6	50	J.S. Bach, "Largo" (Concerto for 2 Violins in D Minor), Hillary Hahn & Margaret Batjer, Bach-Concertos, Los Angeles Chamber Orchestra, Deutsche Grammophon, 474 6392
6	2	Antonio Vivaldi, "Et in terra pax" (Gloria in D Major), Atlanta Symphony, Robert Shaw, Telarc CD-80194
5	57	J.S. Bach, "Komm suesser Tod" (Bach/Stokowski), Full Dimensional Sound, EMI CDM 7243 5 66385 2 5
5	15	W.A. Mozart, "Laudate Dominum", Vesperae solennes de confessore, Kiri Te Kanawa, London Symphony, Sir Colin Davis, Philips 412 873-2
12	4	Max Bruch, "Kol Nidrei", Op. 47, Jacqueline Du Pre & Daniel Barenboim , Brahms Cello Sonatas, EMI

46 minutes, 10 seconds

8	18	Johannes Brahms, "Adagio", Concerto for Violin & Orchestra in D Major, Opus 77, Chicago Symphony, Fritz Reiner, Jascha Heifetz, HMG 09026-61742-2.
9	47	Henryk Gorecki, "Lento e Largo--Tranquillissimo", Symphony No. 3, Op. 36,(Movement #2), Op. cit.
6	33	Sir Edward Elgar, "Larghetto" (Serenade for String Orchestra in E Minor, Op. 20), Halle, Mark Elder, CD HLL 7501.
3	49	Gabriel Faure, "In Paradisum", Requiem, Op.48, Choir of St. John's College, Cambridge, George Guest, London 436-486-2.
7	14	W.A. Mozart, "Adagio", Clarinet Concerto KV622, Jean-Francois Paillard, Jacques Lancelot, Erato 2292-45978-2
6	19	Arvo Part, "Cantus in Memory of Benjamin Britten", from "Sanctuary", Bournemouth Sinfonietta, Richard Studt, Virgin Classics, CSC 7243 5 45314 2 2
6	55	Arvo Part, "Nunc Dimittis", Sabat Mater, Black Box, BBM1071
27	38	El-hadre 2, the Mystik Dance, Klaus Weise, CD-263 (latter half)

©2020 Davis AK et al. *JAMA Psychiatry*.

8	37	Ludwig van Beethoven, "Adagio un poco moto", Piano Concerto #5 ("Emperor"), Leon Fleisher, George Szell, Cleveland Orchestra, Sony "Essential Classics", SBK 46549.
17	37	Russill Paul, "Om Namah Shivaya", Shakti Yoga, The Yoga of Sound, The Relaxation Company, CD 3133
4	3	WA Mozart, "Ave Verum Corpus" (KKV618, London Symphony, Colin Davis, Phillips: 412 873-2.
10	31	Gustav Mahler, "Adagietto" (Symphony #5), Lorin Maazel, Vienna Philharmonic, Sony 696998985025,

1 hour, 57 minutes, 21 seconds

7	47	Alan Hovhaness, "Andante con moto", Symphony #2, "Mysterious Mountain," Seattle Symphony, Gerard Schwarz, Telarc 089408060427.
5	42	Joseph Canteloube, "Bailero" , Songs of the Auvergne, Orchestre de l"opera National de Lyon, Kent Nagano, Dawn Upshaw, Erato 0630-17577-2.
2	10	Richard Strauss, "Moderato", Death and Transfiguration, Andre Previn, Wiener Philharmoniker, Telarc. CD-80167
6	2	Richard Strauss, "Tranquillo", Death and Transfiguration, Ibid.
23	31	Russill Paul, "Evening Shadows Fall", Nada Yoga, The Yoga of Sound, Gaiam, The Relaxation Company, CD 3133.
14	51	J.S. Bach, "Passacaglia & Fugue in C Minor", Bach/Stokowski, Full Dimensional Sound, EMI: CDM 7243 5 66385 2 5.
8	15	Arvo Part, "Spiegel im Spiegel for violin and piano", Sabat Mater, Black Box, BBM1071

1 hour, 8 minutes, 18 sconds

3	3	Enya, "Storms in Africa II", Watermark, Reprise 9 26774-2.
5	38	Guem, "Transe", Musique de Transe, Le Chant Du Monde LDX 2741008
4	1	Adiemus, "Adiemus", Pure Moods, Virgin, 724384218621.
4	22	Gipsy Kings, "Caminando Por la Calle", Mosaique, Nonesuch, 075596089227.
4	27	Mercedes Sosa, "Gracias a La Vida", Gracias a la Vida, Polygram International, 042283231429.
2	21	Louis Armstrong, "What a Wonderful World", What a Wonderful World, Double Play (Intercontinental 600), 607707405826.
60	9	"Ocean Waves", Echos of Nature: The Natural Sounds of the Wilderness, Delta, 018111591621.

1 hour 24 minutes, 01 seconds

Total Duration: 7 hours, 40 minutes, 54 seconds

©2020 Davis AK et al. *JAMA Psychiatry*.

Non-Completing Participants

As shown in the Figure 1 of the study, there were three participants who enrolled in the study and were randomized but did not complete the intervention. For two of these three participants there was no follow-up outcome assessments completed because the study design did not include an intent-to-treat approach. However, for one of the three participants (designated by footnote e in Figure 1), outcome assessments were continued because it was deemed clinically necessary by study personnel to retain contact with this participant. Outcomes from this individual were not included in analyses because he/she only completed one psilocybin session, but data showed that depression scores on the GRID-HAMD decreased from 22 at baseline to a score of 2 at 1 week follow-up and a score of 1 at 4 week follow-up.

Primary Outcomes Assessment Interrater Reliability

Three clinician raters assessed the primary outcome measure for depression (GRID-HAMD) via telephone. All raters were employed at Johns Hopkins and did not conduct any other study assessments nor had any other study involvement. The three raters were trained on a set of standardized and practice interviews developed and conducted by an expert rater (author AKD). Raters were required to be within 3 points of the rating of the expert rater (author AKD) in order to proceed with conducting ratings in the trial. After each rater began rating depression in participants, each of their assessments were rated by another rater using audio recordings until they achieved three consecutively reliable ratings. A rating was considered reliable if the rating from the second rater was within 3 points of the rating by the primary rater. If a rating fell outside of this range, then the two raters met to review and discuss the audio recording and to mutually agree on a final rating. Following initial training and establishing reliability in assessment measurement, ongoing inter-rater reliability was examined for each rater. This examination consisted of randomly selecting one assessment out of every ten assessments for each rater. A second rater listened to the audio recording of the selected assessment. If the rating from the second rater was within 3 points of the rating by the primary rater, the primary rating was used. If the rating was not within 3 points, then the two raters met to review the audio recording and to mutually agree on the final rating.

Assessment of Secondary Outcomes

As a safety precaution, the Columbia Suicide Severity Rating Scale (C-SSRS) was assessed at every in-person visit to assess for potentially worsening suicidality. The "Lifetime/Recent" version of the C-SSRS was used at baseline and the "Since last visit" version for all subsequent administrations. Blood pressure and heart rate were assessed before capsule administration and at 30, 60, 90, 120, 180, 240, 300, and 360 minutes after capsule administration. When psilocybin effects had subsided and at least 7 hours after psilocybin administration, participants completed the Mystical Experience Questionnaire (MEQ30)[1,2] and Challenging Experience Questionnaire (CEQ26).[3] One week following each psilocybin session, participants completed questionnaires developed for assessing persisting effects[4,5] including ratings of the degree of personal meaning, spiritual significance, psychological insight, and psychological challenge attributed to the psilocybin session experience.

Data Analyses

Descriptive statistics for all secondary outcome variables were calculated. Repeated measures ANOVAs were used to examine changes in depression, anxiety, and suicidal ideation scores from baseline to 5 weeks and 8 weeks after enrollment between those in the Immediate

Treatment and Delayed Treatment conditions, and effect sizes were calculated using the partial eta squared statistic. Follow-up independent samples t-tests were used to compare 5 week and 8 week depression, anxiety, and suicidal ideation scores between those in the Immediate Treatment and Delayed Treatment conditions. Overall treatment effects for the entire sample were examined using a series of one-way ANOVAs comparing overall depression, anxiety, and suicidal ideation scores from baseline to 1- and 4-weeks post psilocybin session 2. Effect sizes for the independent samples t-tests and one-way ANOVAs were calculated using the Cohen's d statistic and effect sizes for the repeated measures ANOVA were calculated using the partial eta squared statistic. Confidence intervals are set at 90% for effect sizes calculated using the eta squared statistic and are set at 95% for those calculated using the Cohen's d statistic.[6]

Several participant-rated outcomes were examined as correlates of changes in depression (GRIDHAMD) from baseline to 1- and 4-weeks post-intervention in all study participants (N=24). For these analyses, two change scores were calculated for changes in depression scores from baseline to 1- and 4-week follow-ups by subtracting the follow-up depression score from the baseline score in each participant. Across participants, that depression change score was then correlated with the highest value from Session 1 and Session 2 for each participant of ratings of personal meaning, spiritual significance, psychological insight, and psychological challenge attributed to sessions and of post-session total scores on the Mystical Experience Questionnaire (MEQ30) and the Challenging Experience Questionnaire (CEQ27).

eResults

Session Timing

Following preparation meetings, two psilocybin sessions were administered between one and three weeks apart (mean duration 1.6 weeks). There were no significant differences in the duration of time between psilocybin sessions between those in the DT condition (Mean = 10.91, SD = 3.5; Range = 11) and the IT condition (Mean = 11.15, SD = 3.9; Range = 13), $t(22)=-.16$, p=.874.

Results of Secondary Outcomes

In eTables1-3 and eFigures 1-8 primary and secondary depression and anxiety outcomes are presented, both for the immediate and delayed treatment conditions separately and for the whole group collapsed across both conditions. These tables and figures show that all primary and secondary depression and anxiety outcomes showed a similar pattern of results, with statistically significant differences between conditions and across both conditions after entry into the active intervention.

A measure of suicidal ideation is presented in eTables 1-3 and eFigure 9. As shown, suicidal ideation decreased in both the immediate and delayed treatment groups across time.

eTable 4 shows that participants attributed high levels personal meaning, spiritual significance, psychological challenge, and psychological insight to the psilocybin sessions with statistically higher ratings of personal meaning associated with session 2 compared to session 1. Additionally, 85-90% of participants rated a psilocybin session to be one of the top five most personally meaningful and psychologically insightful experiences of their lives, with 40-60% rating a session to be the single most meaningful, spiritually significant or psychologically insightful of their lives.

©2020 Davis AK et al. *JAMA Psychiatry*.

eTable 5 shows the results from the Mystical Experience Questionnaire and the Challenging Experience Questionnaire assessed at the end of each of two psilocybin sessions. Results indicate that 9 (38%), and 13 (54%), participants had a complete mystical experience (≥ 60% of maximum score on all 4 factors of the MEQ) in the first and second psilocybin session, respectively. Overall, 63% of participants had a complete mystical experience in one or both psilocybin sessions.

Decreases in depression scores at 4-weeks had a significant and moderate-to-strong correlation with the highest score from the two psilocybin sessions on ratings of the degree to which participants reported that their psilocybin sessions were personally meaningful (r=-.70, p<.01), psychological insightful (r=-.60, p<.01), and spiritually significant (r=-.57, p<.01). Having had a complete mystical experience was not significantly associated with changes in depression scores. Decreases in depression scores at 4-weeks had a significant and moderate correlation with the highest score from the two psilocybin sessions on the on the Mystical Experience Questionnaire (MEQ30) (r=-.41, p<.05). Changes in depression were not significantly correlated with comparable ratings of the degree of challenging experiences and scores on the Challenging Experience Questionnaire (CEQ27).

As shown in eTable 6, peak ratings by session facilitators of overall drug effect and distance from reality were statistically higher ratings in session 2 compared to session 1. There were no differences in peak heart rate or blood pressure between session 1 and session 2 (eTable 7).

There were no serious adverse events in the study. Adverse emotional and physical effects during sessions or after sessions are shown in eTables 8 and 9. As shown in eTable 8, more than half the participants endorsed experiencing a variety of potentially unpleasant or challenging emotions and physical sensations during psilocybin sessions, however other studies have shown that such experiences after psilocybin are not uncommon.[3,7] Increases in blood pressure and heartrate above criteria levels were rare and resolved spontaneously. Headache occurred during 33% of sessions (eTable 8) and after 29% of sessions (eTable 9). Elevated rates of post-psilocybin headache have been reported previously.[8]

As shown in eTable 10, at the 4 week follow-up one patient had initiated antidepressant medication, eight had engaged in psychotherapy, and none reported use of psilocybin or psilocybin containing mushrooms. Out of the 8 participants who reported engaging in psychotherapy at the 4 week follow-up, 7 had been continuously engaged in psychotherapy during the course of the study and 1 had initiated an intake/consultation with a new therapist. Lastly, there were no reports of psilocybin or psilocybin mushroom use outside of the trial during the 4-weeks following the intervention.

References

1. MacLean KA, Johnson MW, Griffiths RR. Mystical experiences occasioned by the hallucinogen psilocybin lead to increases in the personality domain of openness. *J Psychopharmacol (Oxf)*. 2011;25(11):1453-1461. doi:10.1177/0269881111420188

2. Barrett FS, Johnson MW, Griffiths RR. Validation of the revised Mystical Experience Questionnaire in experimental sessions with psilocybin. *J Psychopharmacol (Oxf)*. 2015;29(11):1182-1190. doi:10.1177/0269881115609019

©2020 Davis AK et al. *JAMA Psychiatry*.

3. Barrett FS, Bradstreet MP, Leoutsakos J-MS, Johnson MW, Griffiths RR. The Challenging Experience Questionnaire: Characterization of challenging experiences with psilocybin mushrooms. *J Psychopharmacol (Oxf)*. 2016;30(12):1279-1295. doi:10.1177/0269881116678781

4. Griffiths RR, Richards WA, McCann U, Jesse R. Psilocybin can occasion mystical-type experiences having substantial and sustained personal meaning and spiritual significance. *Psychopharmacology (Berl)*. 2006;187(3):268-283. doi:10.1007/s00213-006-0457-5

5. Griffiths RR, Johnson MW, Richards WA, Richards BD, McCann U, Jesse R. Psilocybin occasioned mystical-type experiences: immediate and persisting dose-related effects. *Psychopharmacology (Berl)*. 2011;218(4):649-665. doi:10.1007/s00213-011-2358-5

6. Steiger JH. Beyond the F test: Effect size confidence intervals and tests of close fit in the analysis of variance and contrast analysis. *Psychol Methods*. 2004;9(2):164-182. doi:10.1037/1082-989X.9.2.164

7. Carbonaro TM, Bradstreet MP, Barrett FS, et al. Survey study of challenging experiences after ingesting psilocybin mushrooms: Acute and enduring positive and negative consequences. *J Psychopharmacol (Oxf)*. 2016;30(12):1268-1278. doi:10.1177/0269881116662634

8. Johnson MW, Andrew Sewell R, Griffiths RR. Psilocybin dose-dependently causes delayed, transient headaches in healthy volunteers. *Drug Alcohol Depend*. 2012;123(1-3):132-140. doi:10.1016/j.drugalcdep.2011.10.029

©2020 Davis AK et al. *JAMA Psychiatry*.

eTables

eTable 1. Repeated measures ANOVAs and effect sizes for depression, anxiety, and suicidal ideation outcomes at Baseline, 5 weeks post randomization, and 8 weeks post randomization for the Immediate treatment condition (N=13) and the Delayed Treatment condition (N=11); the 5-week and 8-week time points correspond to 1-week and 4-weeks post session 2 in the Immediate Treatment condition. There were no missing data.

Measure (condition)	Baseline 0 weeks M(SD)	Time 2 5 weeks M(SD)	Time 3 8 weeks M(SD)	Time F-stat[a]	Time Effect Size (η^2)[b] (90% CI)	Condition F-stat[a]	Condition Effect Size (η^2)[b] (90% CI)	Time by Condition F-stat[a]	Time by Condition Effect Size (η^2)[b] (90% CI)
**Depression Measures**									
GRID-HAMD				21.3***	.49^^^	32.6***	.60^^^	29.1***	.57^^^
Immediate	22.9 (3.6)	8.0 (7.1)	8.5 (5.7)		(.29; .60)		(.33; .72)		(.38; .66)
Delayed	22.5 (4.4)	23.8 (5.4)	23.5 (6.0)						
QIDS-SR				36.5***	.63^^^	86.5***	.80^^^	112.6***	.84^^^
Immediate	16.2 (3.6)	5.2 (4.6)	5.5 (3.6)		(.46; .72)		(.64; .86)		(.75; .88)
Delayed	17.4 (3.6)	31.9 (5.6)	17.7 (3.7)						
BDI-II				48.6***	.69^^^	38.4***	.64^^^	58.5***	.73^^^
Immediate	31.9 (7.0)	8.2 (8.9)	8.2 (7.0)		(.54; .76)		(.39; .75)		(.59; .79)
Delayed	34.5 (10.0)	35.2 (9.1)	35.9 (8.4)						
PHQ-9[c]				39.9***	.66^^^	45.6***	.68^^^	54.2***	.72^^^
Immediate	16.5 (3.1)	-	4.8 (2.9)		(.41; .76)		(.45; .78)		(.50; .81)
Delayed	17.9 (3.3)	-	18.8 (4.3)						
**Anxiety Measures**									
HAM-A				8.0*	.27^^^	27.0***	.55^^^	32.3***	.59^^^
Immediate	17.1 (4.1)	-	6.8 (3.9)		(.04; .47)		(.28; .69)		(.33; .72)
Delayed	17.9 (4.3)	-	21.4 (6.3)						
STAI – State				8.5**	.28^^^	23.8***	.52^^^	22.1***	.50^^^
Immediate	45.5 (10.3)	-	33.5 (6.6)		(.05; .48)		(.25; .66)		(.23; .65)
Delayed	52.7 (6.6)	-	55.5 (8.8)						
STAI – Trait				7.0*	.24^^^	6.3**	.22^^^	30.8***	.58^^^
Immediate	59.0 (7.2)	-	44.9 (10.1)		(.03; .45)		(.02; .43)		(.32; .71)
Delayed	58.0 (10.5)	-	63.0 (9.3)						

©2020 Davis AK et al. *JAMA Psychiatry*.

Measure (condition)	Baseline 0 weeks M(SD)	Time 2 5 weeks M(SD)	Time 3 8 weeks M(SD)	Time F-stat[a]	Time Effect Size (η^2)[b] (90% CI)	Condition F-stat[a]	Condition Effect Size (η^2)[b] (90% CI)	Time by Condition F-stat[a]	Time by Condition Effect Size (η^2)[b] (90% CI)
STAI – Total				9.0**	.29^^^	17.1***	.44^^^	30.9***	.58^^^
Immediate	104.5 (15.1)	-	78.5 (14.8)		(.05; .49)		(.16; .60)		(.32; .71)
Delayed	110.7 (16.3)	-	118.5 (16.1)						
**Suicidal Ideation Measure**									
CSSRS				14.1***	.39^^^	1.1	-	.7	-
Immediate	1.2 (1.2)	0.2 (0.4)	0.2 (0.4)		(.18; .52)				
Delayed	1.3 (1.3)	0.6 (0.9)	0.5 (0.9)						

[a] *p<.05, **p<.01, ***p<.001
[b] Effect sizes are shown when F-tests are significant; ^small effect (>.01), ^^medium effect (>.06), ^^^large effect (>.14)
[c] One participant did not complete the PHQ-9; total sample size for this analysis is n=23.
M: mean; SD: standard deviation
CI: Confidence Interval
GRID-HAMD: GRID-Hamilton Depression Rating Scale. Range of scores 0-52; higher scores indicate more severe depression
QIDS-SR: Quick Inventory of Depression Symptoms. Range of scores 0-27; higher scores indicate more severe depression
PHQ-9: Patient Health Questionnaire – 9 item. Range of scores 0-27; higher scores indicate more severe depression
BDI-II: Beck Depression Inventory – II. Range of scores 0-63; higher scores indicate more severe depression
HAM-A: Hamilton Anxiety Scale. Range of scores 0-56; higher scores indicate more severe anxiety
STAI-State: State-Trait Anxiety Inventory – State Scale. Range of scores 0-80; higher scores indicate greater levels of State anxiety
STAI-Trait: State-Trait Anxiety Inventory – Trait Scale. Range of scores 0-80; higher scores indicate greater levels of Trait anxiety
STAI-Total: State-Trait Anxiety Inventory – Total Scale. Range of scores 0-160; higher scores indicate greater anxiety
CSSRS: Columbia Suicide Severity Rating Scale. Highest level of suicidal ideation is reported. Range of scores 0-5; higher scores indicate greater degree of suicidal ideation

eTable 2. t-tests and effect sizes for depression, anxiety, and suicidal ideation outcomes at Baseline, 5 weeks post randomization and 8 weeks post randomization for the Immediate treatment condition (N=13) and the Delayed Treatment condition (N=11); the 5-week and 8-week time points correspond to 1-week and 4-weeks post session 2 in the Immediate Treatment condition. There were no missing data.

Measures (Timepoint)	Immediate Treatment M (SD)	Delayed Treatment M (SD)	t-test[a]	Effect Size[b] Cohen's d (95% CI)
**Depression**				
GRID-HAMD				
Baseline	22.9 (3.6)	22.5 (4.4)	-.2	-
Time 2 (5wk post-randomization)	8.0 (7.1)	23.8 (5.4)	6.0^{***}	2.5 (95%CI: 1.4; 3.5)$^{\wedge\wedge\wedge}$
Time 3 (8wk post-randomization)	8.5 (5.7)	23.5 (6.0)	6.3^{***}	2.6 (95%CI: 1.5; 3.7)$^{\wedge\wedge\wedge}$
QIDS-SR				
Baseline	16.2 (3.6)	17.3 (3.4)	.8	-
Time 2 (5wk post-randomization)	5.2 (4.6)	31.5 (5.5)	12.5^{***}	5.2 (95%CI: 3.5; 7.0)$^{\wedge\wedge\wedge}$
Time 3 (8wk post-randomization)	5.5 (3.6)	17.7 (3.7)	8.0^{***}	3.4 (95%CI: 2.1; 4.7)$^{\wedge\wedge\wedge}$
BDI-II				
Baseline	31.9 (7.0)	34.5 (10.0)	.7	-
Time 2 (5wk post-randomization)	8.2 (8.9)	35.2 (9.1)	7.3^{***}	3.0 (95%CI: 1.8; 4.2)$^{\wedge\wedge\wedge}$
Time 3 (8wk post-randomization)	8.2 (7.0)	35.9 (8.4)	8.9^{***}	3.6 (95%CI: 2.3; 4.9)$^{\wedge\wedge\wedge}$
PHQ-9				
Baseline	16.5 (3.1)	17.9 (3.3)	1.1	-
Time 3 (8wk post-randomization)	4.8 (2.9)	18.6 (4.2)	9.5^{***}	3.9 (95%CI: 2.5; 5.3)$^{\wedge\wedge\wedge}$
**Anxiety**				
HAM-A				
Baseline	17.1 (4.1)	17.9 (4.3)	.5	-
Time 3 (8wk post-randomization)	6.8 (3.9)	21.4 (6.3)	6.9^{***}	2.8 (95%CI: 1.7; 4.0)$^{\wedge\wedge\wedge}$
STAI – State				
Baseline	45.5 (10.3)	52.7 (6.6)	2.0	-
Time 3 (8wk post-randomization)	33.5 (6.6)	55.5 (8.8)	7.0^{***}	2.9 (95%CI: 1.7; 4.0)$^{\wedge\wedge\wedge}$
STAI – Trait				
Baseline	59.0 (7.2)	58.0 (10.5)	.3	-
Time 3 (8wk post-randomization)	44.9 (10.1)	63.0 (9.3)	4.5^{***}	1.9 (95%CI: 0.9; 2.8)$^{\wedge\wedge\wedge}$

©2020 Davis AK et al. *JAMA Psychiatry*.

Measures (Timepoint)	Immediate Treatment M (SD)	Delayed Treatment M (SD)	t-test[a]	Effect Size[b] Cohen's d (95% CI)
STAI – Total				
Baseline	104.5 (15.1)	110.7 (16.3)	1.0	-
Time 3 (8wk post-randomization)	78.5 (14.8)	118.5 (16.1)	6.4***	2.6 (95%CI: 1.5; 3.7)^^^
**Suicidal Ideation**				
CSSRS				
Baseline	1.2 (1.2)	1.3 (1.3)	.1	-
Time 2 (5wk post-randomization)	0.2 (0.4)	0.6 (0.9)	1.7	-
Time 3 (8wk post-randomization)	0.2 (0.4)	0.5 (0.9)	1.4	-

[a] *p<.05, **p<.01, ***p<.001
[b] Effect sizes are shown when t-tests are significant; ^small effect (>.30), ^^medium effect (>.50), ^^^large effect (>.80)
M: mean; SD: standard deviation
CI: Confidence Interval
GRID-HAMD: GRID-Hamilton Depression Rating Scale. Range of scores 0-52; higher scores indicate more severe depression
QIDS-SR: Quick Inventory of Depression Symptoms. Range of scores 0-27; higher scores indicate more severe depression
PHQ-9: Patient Health Questionnaire – 9 item. Range of scores 0-27; higher scores indicate more severe depression
BDI-II: Beck Depression Inventory – II. Range of scores 0-63; higher scores indicate more severe depression
HAM-A: Hamilton Anxiety Scale. Range of scores 0-56; higher scores indicate more severe anxiety
STAI-State: State-Trait Anxiety Inventory – State Scale. Range of scores 0-80; higher scores indicate greater levels of State anxiety
STAI-Trait: State-Trait Anxiety Inventory – Trait Scale. Range of scores 0-80; higher scores indicate greater levels of Trait anxiety
STAI-Total: State-Trait Anxiety Inventory – Total Scale. Range of scores 0-160; higher scores indicate greater anxiety
CSSRS: Columbia Suicide Severity Rating Scale. Highest level of suicidal ideation is reported. Range of scores 0-5; higher scores indicate greater degree of suicidal ideation

©2020 Davis AK et al. *JAMA Psychiatry*.

eTable 3. Repeated measures ANOVAs and effect sizes for depression, anxiety, and suicidal ideation outcomes at Baseline, 5 weeks post randomization, and 8 weeks post randomization for the overall sample (N=24); the 5-week and 8-week time points correspond to 1-week and 4-weeks post session 2 in the Immediate Treatment condition. There were no missing data.

Measures	Baseline 0 weeks M(SD)	Time 2 5 weeks M(SD)	Time 3 8 weeks M(SD)	F-stat[a]	Effect Size η^{2b} (90% CI)
**Depression**					
GRID-HAMD	22.8 (3.9)	8.7 (7.6)†	8.9 (7.4)†	75.5***	.77^^ (.65; .82)
QIDS-SR	16.7 (3.5)	5.8 (5.4)†	6.0 (5.7)†	69.0***	.75^^ (.63; .81)
BDI-II	33.1 (8.4)	10.1 (11.4)†	9.3 (11.3)†	87.3***	.79^^ (.69; .84)
PHQ-9	17.1 (3.2)	-	5.2 (5.0)†	111.5***	.84^^^ (.70; .88)
**Anxiety**					
HAM-A	17.5 (4.1)	-	7.8 (6.8)†	44.0***	.66^^ (.42; .76)
STAI – State	48.8 (9.3)	-	35.0 (12.8)†	32.3***	.58^^ (.33; .71)
STAI – Trait	58.5 (8.7)	-	44.3 (13.1)†	28.2***	.55^^ (.29; .68)
STAI – Total	107.4 (15.7)	-	79.2 (24.8)†	32.4***	.58^^ (.33; .71)
**Suicidal Ideation**					
CSSRS	1.3 (1.2)	.3 (.7)†	.2 (.7)†	18.0***	.44^ (.24; .56)

[a] *p<.05, **p<.01, ***p<.001
[b] Effect sizes are shown when F-tests are significant; ^small effect (>.01), ^^medium effect (>.06), ^^^large effect (>.14)
† Indicates that value was significantly difference from Baseline; values with the same symbol in each row are not statistically different from one another using post-hoc mean pairwise comparisons with Bonferroni correction
M: mean; SD: standard deviation
CI: Confidence Interval
GRID-HAMD: GRID-Hamilton Depression Rating Scale. Range of scores 0-52; higher scores indicate more severe depression
QIDS-SR: Quick Inventory of Depression Symptoms. Range of scores 0-27; higher scores indicate more severe depression
PHQ-9: Patient Health Questionnaire – 9 item. Range of scores 0-27; higher scores indicate more severe depression
BDI-II: Beck Depression Inventory – II. Range of scores 0-63; higher scores indicate more severe depression
HAM-A: Hamilton Anxiety Scale. Range of scores 0-56; higher scores indicate more severe anxiety
STAI-State: State-Trait Anxiety Inventory – State Scale. Range of scores 0-80; higher scores indicate greater levels of State anxiety
STAI-Trait: State-Trait Anxiety Inventory – Trait Scale. Range of scores 0-80; higher scores indicate greater levels of Trait anxiety
STAI-Total: State-Trait Anxiety Inventory – Total Scale. Range of scores 0-160; higher scores indicate greater anxiety
CSSRS: Columbia Suicide Severity Rating Scale. Highest level of suicidal ideation is reported. Range of scores 0-5; higher scores indicate greater degree of suicidal ideation

©2020 Davis AK et al. *JAMA Psychiatry*.

eTable 4. Participant ratings of personal meaning, spiritual significance, psychological challenge, and psychological insight attributed to the psilocybin sessions. Ratings occurred 1-day post psilocybin session 1 and 1-day post psilocybin session 2 [a,b]

Psilocybin Session (rating)	Highest Rating M(SD)[c]	Psilocybin Session 1 M(SD) or n(%)	Psilocybin Session 2 M(SD) or n(%)	z- or t-stat[d]
Overall Rating				
Personally Meaningful	7.1 (1.2)	6.3 (1.5)	7.1 (1.2)	-3.11**
Spiritually Significant	6.6 (2.5)	5.7 (2.8)	6.2 (2.7)	-.90
Psychologically Challenging	6.3 (2.0)	5.6 (2.2)	5.6 (2.5)	.00
Psychologically Insightful	7.3 (1.4)	6.9 (1.8)	7.1 (1.3)	-.81
Top 5 including single most				
Personally Meaningful	17 (85%)	12 (60%)	17 (85%)	-1.77
Spiritually Significant	15 (75%)	11 (55%)	13 (65%)	-.65
Psychologically Challenging	13 (65%)	11 (55%)	10 (50%)	.32
Psychologically Insightful	18 (90%)	16 (80%)	18 (90%)	-.89
Single Most				
Personally Meaningful	8 (40%)	4 (20%)	8 (40%)	-1.38
Spiritually Significant	12 (60%)	8 (40%)	10 (50%)	-.64
Psychologically Challenging	6 (25%)	3 (15%)	4 (20%)	-.42
Psychologically Insightful	12 (60%)	11 (55%)	8 (40%)	.95

[a] Only 20 (of 24) participants completed these measures due to an error in the survey programming
[b] Rating options ranged from 1=no more than routine, everyday experiences to 7=among the 5 most [meaningful, spiritually significant, psychologically insightful, or difficulty/psychologically challenging] of my life, and 8=the single most [meaningful, spiritually significant, psychologically insightful, or difficulty/psychologically challenging] of my life.
[c] Data shown in this column reflect the highest rating from Session 1 and 2 for each participant
[d] Statistical comparison between Session 1 and Session 2; **p<.01
M: mean; SD: standard deviation

©2020 Davis AK et al. *JAMA Psychiatry*.

eTable 5. Results from the Mystical Experience Questionnaire and The Challenging Experience Questionnaire assessed at the end of each of two psilocybin sessions. Data are the mean proportion (and standard deviation) of total possible score and the percentage of participants who fulfilled criteria for complete mystical experience (N=24). There were no missing data.

Outcome Measure	Highest Rating M (SD) or %	Psilocybin Session 1 (20 mg/70 kg) M (SD) or %	Psilocybin Session 2 (30 mg/70 kg) M (SD) or %	z- or t-stat[a]
**Mystical Experience Questionnaire**				
Mystical Experience	.71 (.3)	.57 (.3)	.65 (.3)	-1.51
Positive Mood	.73 (.3)	.64 (.3)	.67 (.3)	-.75
Transcendence	.75 (.3)	.63 (.3)	.68 (.3)	-.94
Ineffability	.88 (.2)	.79 (.2)	.84 (.2)	-1.37
Total Score	.73 (.3)	.62 (.3)	.68 (.3)	-1.41
Complete Mystical Experience	63%	38%	54%	-1.11
**Challenging Experience Questionnaire**				
Fear	.42 (.3)	.31 (.3)	.33 (.3)	-.30
Grief	.67 (.2)	.59 (.2)	.47 (.3)	1.69
Physical Distress	.49 (.2)	.39 (.2)	.41 (.2)	-.50
Insane	.34 (.3)	.24 (.3)	.22 (.3)	.29
Isolation	.58 (.3)	.43 (.3)	.35 (.3)	.83
Feel like dead or dying	.34 (.4)	.21 (.3)	.22 (.3)	-.16
Paranoia	.13 (.3)	.06 (.1)	.12 (.3)	-1.46
Total Score	.45 (.2)	.37 (.2)	.34 (.2)	.51

M: mean; SD: standard deviation
[a] Comparison of Session 1 with Session 2 data. All of these tests were non-significant. t-tests results are shown for ratings on Mystical Experience and Challenging Experience Questionnaires. Results of a two-proportion z-test is shown to compare the percentage of participants that fulfilled criteria for having had a complete mystical experience.

©2020 Davis AK et al. *JAMA Psychiatry*.

eTable 6. Means (and standard deviations) of peak ratings during the session by both session facilitators for each of two psilocybin sessions (N=24). There were no missing data.

Outcome Measure	Session 1 M(SD)	Session 2 M(SD)	t-stat
Overall Drug Effect	2.5 (0.5)	3.0 (0.4)	-3.73**
Anxiety/fear	1.3 (0.6)	1.4 (0.9)	-1.16
Distance from reality	2.3 (0.6)	2.8 (0.6)	-3.35**
Systematized Delusions	0.0 (0.2)	0.0 (0.1)	.00
Yawning	0.9 (1.2)	1.0 (1.2)	-.81
Tears/Crying	2.1 (1.0)	1.8 (1.3)	1.33
Nausea/Vomiting	0.5 (0.7)	0.4 (0.6)	.68
Joy/Intense Happiness	1.5 (1.1)	1.8 (1.1)	-1.33
Peace/Harmony	1.8 (1.0)	2.2 (0.9)	-2.02
Psychological Discomfort	1.2 (0.7)	1.3 (1.0)	-.74
Psychological Distress	0.6 (1.0)	0.7 (0.9)	-.51

M: mean; SD: standard deviation
**p<.01
Note. Maximum possible rating for these monitor rated outcome measures were 4. Peak effects were defined as the maximum value observed during the session after drug administration for each participant. Data are means of the peak rating from both facilitators.

©2020 Davis AK et al. *JAMA Psychiatry*.

eTable 7. Means (and standard deviations) of peak heart rate and blood pressure during each of two psilocybin sessions (N=24). There were no missing data.

Outcome Measure	Session 1<br>M(SD)	Session 2<br>M(SD)	t-stat[a]
Heartrate	86.6 (16.3)	86.0 (13.1)	.25
Systolic	138.7 (13.0)	140.0 (10.7)	-.93
Diastolic	87.6 (9.1)	87.1 (7.2)	.30

[a] All t-statistics were non-significant
M: mean; SD: standard deviation
Note. Peak effects were defined as the maximum value observed during the session after drug administration for each participant.

eTable 8. Adverse emotional and physical effects during psilocybin sessions. There were no missing data.

Measure	Total number across both sessions n (% of 48)	M (SD)	Session 1 n (% of 24)	M(SD)	Session 2 n (% of 24)	M(SD)
**Endorsed on the Challenging Experiences Questionnaire at end of the session**[a]						
I felt like crying	44 (92%)	3.7 (1.0)	24 (100%)	3.6 (.9)	20 (83%)	3.8 (1.0)
Sadness	38 (79%)	3.8 (.8)	21 (88%)	4.0 (.9)	17 (71%)	3.5 (.8)
Emotional and/or physical suffering	37 (77%)	3.8 (.8)	21 (88%)	3.7 (.8)	16 (67%)	3.9 (.7)
Feeling my heart beating	34 (71%)	2.9 (.9)	17 (71%)	2.8 (.9)	17 (71%)	3.1 (1.0)
Feeling my body shake/tremble	32 (67%)	3.3 (1.1)	16 (67%)	3.2 (1.0)	16 (67%)	3.3 (1.2)
Pressure or weight in my chest or abdomen	32 (67%)	3.5 (1.0)	16 (67%)	3.3 (.9)	16 (67%)	3.8 (1.1)
I felt shaky inside	30 (63%)	3.4 (1.0)	16 (67%)	3.3 (.9)	14 (58%)	3.6 (1.1)
Feelings of grief	29 (60%)	3.9 (.7)	17 (71%)	4.1 (.6)	12 (50%)	3.7 (.9)
Isolation and loneliness	28 (58%)	3.7 (.9)	15 (63%)	3.7 (.9)	13 (54%)	3.7 (.9)
Despair	28 (58%)	3.4 (1.0)	14 (58%)	3.4 (1.1)	14 (58%)	3.4 (1.0)
Anxiousness	27 (56%)	3.3 (1.0)	13 (54%)	3.2 (.9)	14 (58%)	3.5 (1.1)
Feeling of isolation from people and things	26 (54%)	3.4 (1.0)	14 (58%)	3.4 (1.0)	12 (50%)	3.3 (1.1)
Experience of fear	26 (54%)	3.1 (1.0)	12 (50%)	3.3 (1.0)	14 (58%)	2.9 (1.0)
Feelings of despair	23 (48%)	3.6 (.9)	12 (50%)	3.5 (1.1)	11 (46%)	3.7 (.8)
I felt isolated from everything and everyone	22 (46%)	3.3 (1.1)	12 (50%)	3.3 (1.2)	10 (42%)	3.2 (1.0)
I felt frightened	21 (44%)	3.1 (.9)	12 (50%)	2.8 (.8)	9 (38%)	3.3 (1.0)
Panic	19 (40%)	3.4 (1.1)	8 (33%)	3.3 (1.2)	11 (46%)	3.5 (1.0)
I had the feeling something horrible would happen	16 (33%)	3.4 (.9)	9 (38%)	3.0 (.9)	7 (29%)	3.7 (1.0)
I was afraid that the state I was in would last forever	15 (31%)	3.6 (1.0)	9 (38%)	3.4 (1.0)	6 (25%)	3.7 (1.0)
I had the profound experience of my own death	15 (31%)	3.3 (1.2)	8 (33%)	3.0 (1.2)	7 (29%)	3.6 (1.1)
I experienced a decreased sense of sanity	14 (29%)	3.9 (.9)	8 (33%)	3.5 (1.1)	6 (25%)	4.3 (.8)
Fear that I might lose my mind or go insane	14 (29%)	3.2 (1.1)	6 (25%)	3.5 (1.0)	8 (33%)	2.9 (1.1)
I felt my heart beating irregularly or skipping beats	12 (25%)	3.1 (.9)	5 (21%)	3.0 (.7)	7 (29%)	3.1 (1.1)
I felt as if I was dead or dying	12 (25%)	3.7 (1.0)	6 (25%)	3.3 (1.0)	6 (25%)	4.0 (.9)
Experience of antagonism toward people around me	8 (17%)	3.0 (1.0)	3 (13%)	2.7 (.6)	5 (21%)	3.4 (1.3)
I had the feeling that people were plotting against me	4 (8%)	3.3 (1.1)	2 (8%)	2.5 (.7)	2 (8%)	4.0 (1.4)

©2020 Davis AK et al. *JAMA Psychiatry*.

Measure	Total number across both sessions n (% of 48)	M (SD)	Session 1 n (% of 24)	M(SD)	Session 2 n (% of 24)	M(SD)
**Cardiovascular and headache events during the session**						
Blood pressure event[b]	1 (2%)	-	1 (4%)	-	0 (0%)	
Heartrate event[c]	4 (8%)		2 (8%)		2 (8%)	
Headache	16 (33%)	-	11 (46%)	-	5 (21%)	

M: mean; SD: standard deviation

[a] Description of item rated. Scores could range from 0-5, with 0=no; not at all; 1= so slight I cannot decide; 2=slight; 3=moderate; 4=strong; 5=extreme (more than ever before in my life). A participant was counted as having the experience if they rated an item on the Challenging Experiences Questionnaire as a 2 or higher.

[b] A blood pressure event was defined by systolic blood pressure (SBP) > 170 mmHG or diastolic blood pressure (DBP) > 100 mmHg. In these instances, assessments were repeated every 5 minutes until criteria were no longer exceeded. If SBP > 200 or DBP > 110, then medical intervention was required, but that never occurred in this study. For the single event in this study, the participant's DBP exceeded the threshold on two occasions. The first at +180min (DBP = 108) and the second at +360min (DBP = 104). On both occasions the blood pressure decreased below the criterion level after 5 minutes.

[c] A heartrate event was defined by heart rate (HR) > 110 beats per minute. In these instances, assessments were repeated every 5 minutes until criteria were no longer exceeded. In three participants, there were a total of four sessions during which criteria for repeated assessment due to elevated heart rate (>110 BPM) were met. On those sessions, additional heartrate assessments were required 1, 2, 14, or 40 times before heartrate returned to below criteria levels. None of the heart rate increases exceeded the maximum heart rate which would have required medical intervention.

©2020 Davis AK et al. *JAMA Psychiatry*.

eTable 9. Adverse effects reported within two weeks after Sessions 1 and 2 that were rated by staff as possibly or probably related to psilocybin[a]. There were no missing data.

Outcome Measure	Session 1<br>n (%)	Session 2<br>n (%)
**After the session**		
Headache	7 (29%)	7 (29%)
Physical Discomfort	1 (4%)	0 (0%)
Mild controllable muscle motion	1 (4%)	0 (0%)
Visual distortion	1 (4%)	2 (8%)
Tenseness/soreness	0 (0%)	2 (8%)
Chest tightness	0 (0%)	1 (4%)
Vivid dreams	0 (0%)	1 (4%)
Altered body sensation	0 (0%)	1 (4%)

[a] Participants were explicitly questioned about the occurrence of headache on the day after each psilocybin sessions. Other instances of headache and other adverse effects were prompted throughout the study by asking the participant if adverse events had occurred since their last visit. Most (88%) of these adverse effects were reported on the day following the psilocybin session.

©2020 Davis AK et al. *JAMA Psychiatry*.

eTable 10. Initiation of antidepressant medication, psychotherapy, or psilocybin use (assessed at 4 week follow-up; N=24). There were no missing data.

Variable	n(%)
Antidepressant Medications	1 (4%)
Psychotherapy	8 (33%)
Psilocybin or Psilocybin Mushrooms	0 (0%)

©2020 Davis AK et al. *JAMA Psychiatry*.

eFigures

eFigure 1. Decrease in depression scores on the Quick Inventory of Depression Symptoms (QIDS-SR) from Baseline to 1 day post psilocybin session 1 through 4 weeks post psilocybin session 2. There were no missing data. Data points are means; brackets show ±1 SD for all participants (N=24). Effect sizes (Cohen's d with 95% confidence interval (CI)) and p-values are from paired sample t-tests comparing scores at Baseline to 1 day post session 1 (d=2.6 [95% CI: 1.8; 3.5]), 1 week post session 1 (d=2.0 [95% CI: 1.3; 2.7]), 1 day post session 2 (d=2.6 [95% CI: 1.8; 3.4]), 1 week post session 2 (d=2.4 [95% CI: 1.6; 3.1]), and 4 weeks post session 2 (d=2.3 [95% CI: 1.5; 3.0]).

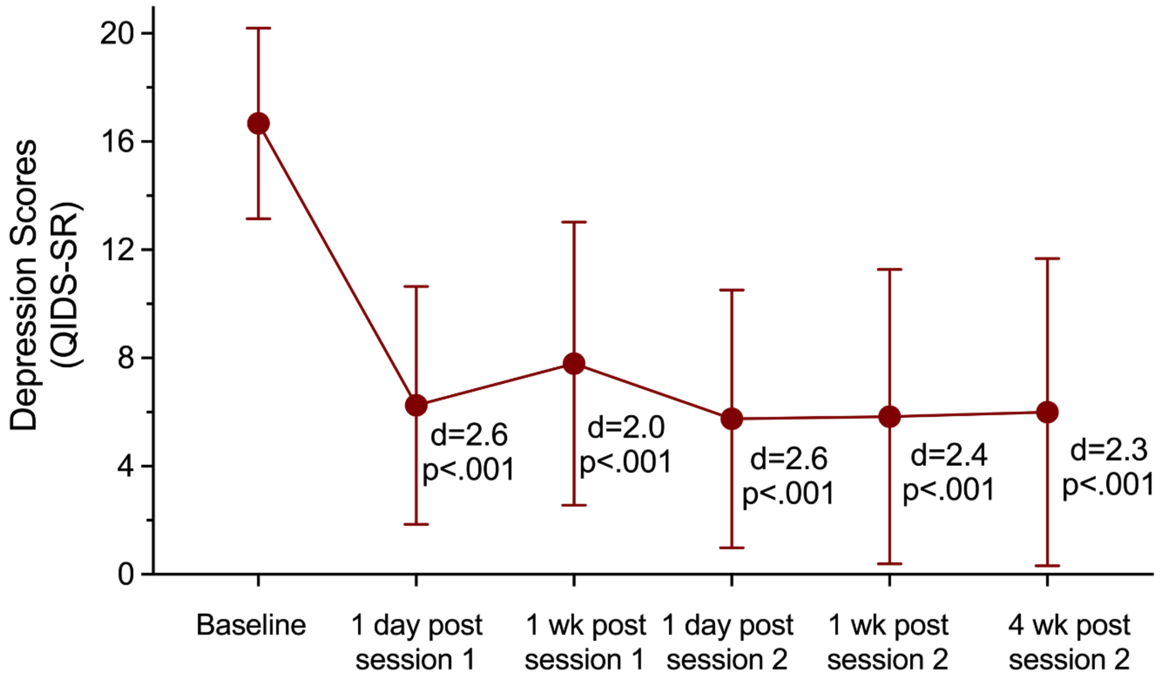

©2020 Davis AK et al. *JAMA Psychiatry*.

eFigure 2. Comparison of depression scores on the Quick Inventory of Depression Symptoms (QIDS-SR) for the Delayed Treatment (n=11) and Immediate Treatment (n=13) conditions. Data points are means; brackets show ±1 SD. There were no missing data. Data are shown for Baseline (Screening) and study weeks 5 and 8. In the Immediate Treatment condition, study weeks 5 and 8 correspond to 1 week and 4 weeks after the psilocybin session 2. In the Delayed Treatment group weeks 5 and 8 are pre-psilocybin assessments obtained during the delay period. Effect size (Cohen's d with 95% confidence interval (CI)) and p-values reflect the results of a two-sample t-test between groups at study weeks 5 (d=5.2 [95% CI: 3.5; 7.0]) and 8 (d=3.4 [95% CI: 2.1; 4.7]).

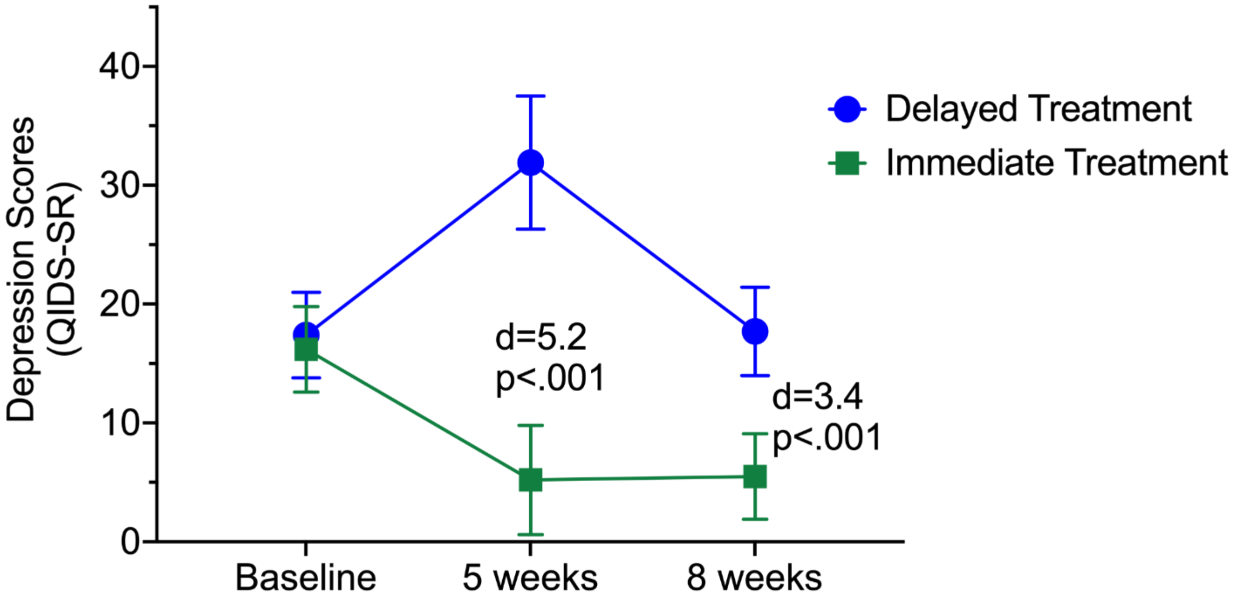

©2020 Davis AK et al. *JAMA Psychiatry*.

eFigure 3. Comparison of depression scores on the Beck Depression Inventory–II (BDI-II) for the Delayed Treatment (n=11) and Immediate Treatment (n=13) conditions. Data points are means; brackets show ±1 SD. There were no missing data. Data are shown for Baseline (Screening) and study weeks 5 and 8. In the Immediate Treatment condition, study weeks 5 and 8 correspond to 1 week and 4 weeks after the psilocybin session 2. In the Delayed Treatment group weeks 5 and 8 are pre-psilocybin assessments obtained during the delay period. Effect size (Cohen's d with 95% confidence interval (CI)) and p-values reflect the results of a two-sample t-test between groups at study weeks 5 (d=3.0 [95% CI: 1.8; 4.2]) and 8 (d=3.6 [95% CI: 2.3; 4.9]).

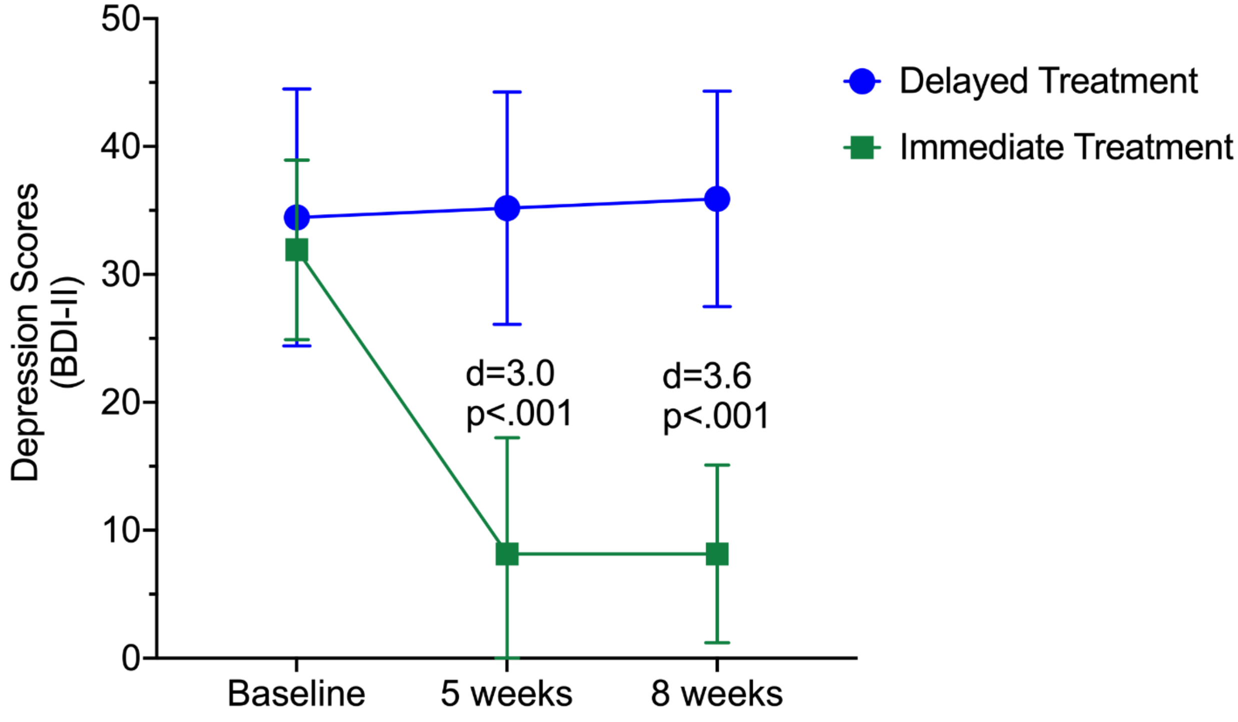

eFigure 4. Comparison of depression scores on the Patient Health Questionnaire–9 item (PHQ-9) for the Delayed Treatment (n=11) and Immediate Treatment (n=13) conditions. Data points are means; brackets show ±1 SD. There were no missing data. Data are shown for Baseline (Screening) and study week 8. In the Immediate Treatment condition, study weeks 8 corresponds to 4 weeks after the psilocybin session 2. In the Delayed Treatment group week 8 is a pre-psilocybin assessment obtained during the delay period. Effect size (Cohen's d with 95% confidence interval (CI)) and p-values reflect the results of a two-sample t-test between groups at study week 8 (d=3.9 [95% CI: 2.5; 5.3]).

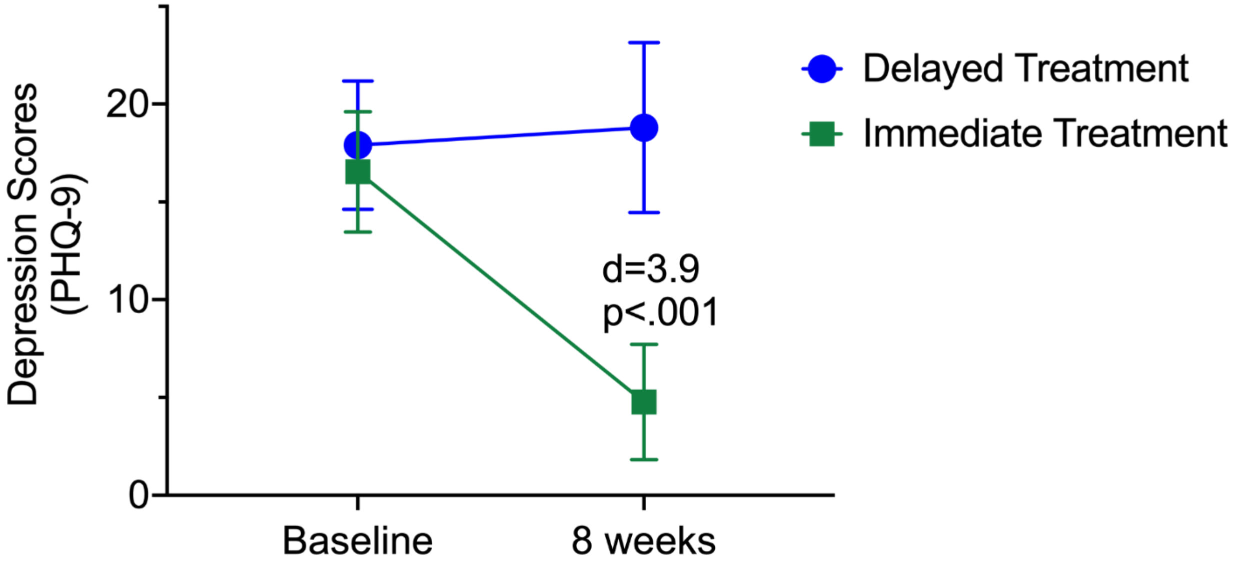

©2020 Davis AK et al. *JAMA Psychiatry*.

eFigure 5. Comparison of anxiety scores on the Hamilton Anxiety Scale (HAM-A) for the Delayed Treatment (n=11) and Immediate Treatment (n=13) conditions. There were no missing data. Data points are means; brackets show ±1 SD. Data are shown for Baseline (Screening) and study week 8. In the Immediate Treatment condition, study weeks 8 corresponds to 4 weeks after the psilocybin session 2. In the Delayed Treatment group week 8 is a pre-psilocybin assessment obtained during the delay period. Effect size (Cohen's d with 95% confidence interval (CI)) and p-values reflect the results of a two-sample t-test between groups at study week 8 (d=2.8 [95% CI: 1.7; 4.0]).

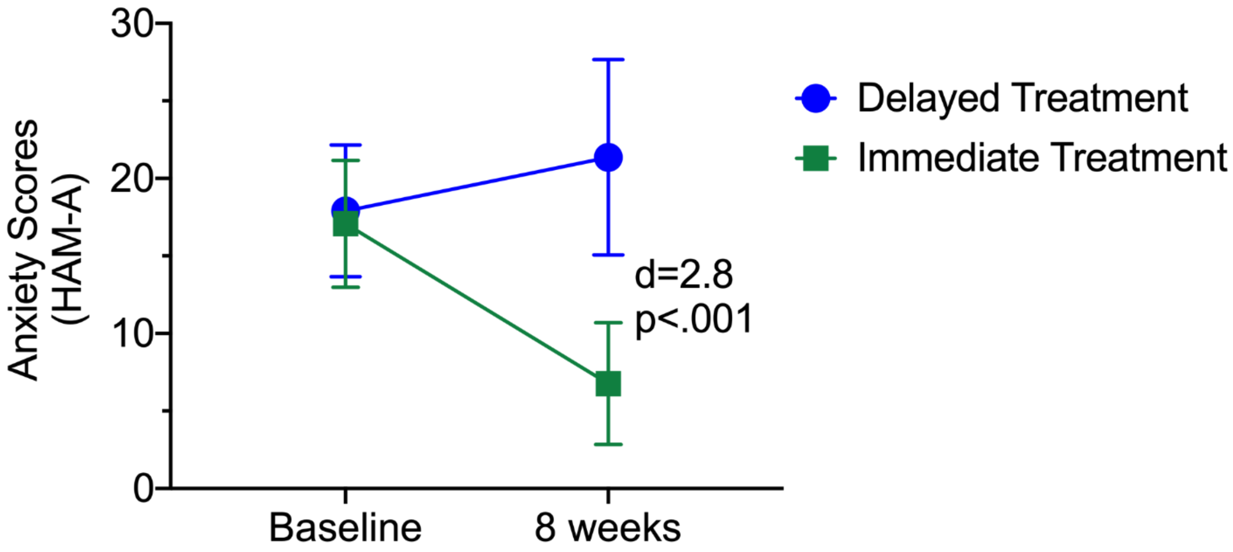

©2020 Davis AK et al. *JAMA Psychiatry*.

eFigure 6. Comparison of anxiety scores on the State-Trait Anxiety Inventory–State Subscale (STAI-State) for the Delayed Treatment (n=11) and Immediate Treatment (n=13) conditions. Data points are means; brackets show ±1 SD. There were no missing data. Data are shown for Baseline (Screening) and study week 8. In the Immediate Treatment condition, study weeks 8 corresponds to 4 weeks after the psilocybin session 2. In the Delayed Treatment group week 8 is a pre-psilocybin assessment obtained during the delay period. Effect size (Cohen's d with 95% confidence interval (CI)) and p-values reflect the results of a two-sample t-test between groups at study week 8 (d=2.9 [95% CI: 1.7; 4.0]).

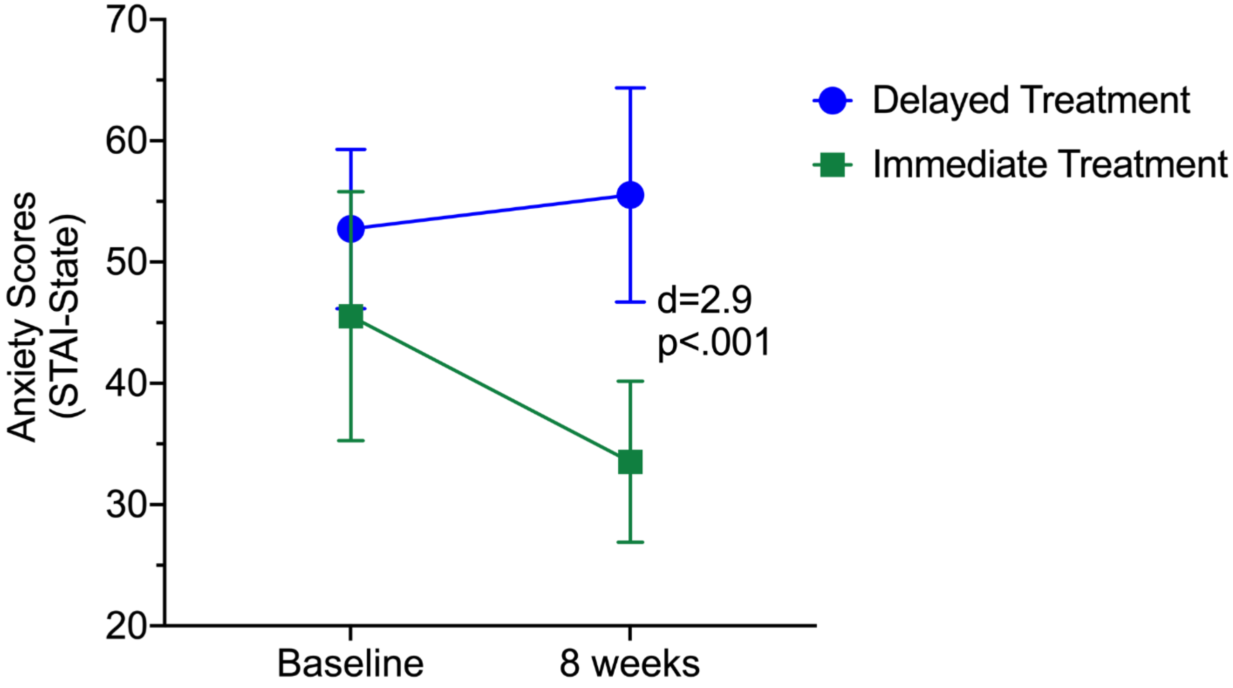

©2020 Davis AK et al. *JAMA Psychiatry*.

eFigure 7. Comparison of anxiety scores on the State-Trait Anxiety Inventory–Trait Subscale (STAI-Trait) for the Delayed Treatment (n=11) and Immediate Treatment (n=13) conditions. Data points are means; brackets show ±1 SD. There were no missing data. Data are shown for Baseline (Screening) and study week 8. In the Immediate Treatment condition, study weeks 8 corresponds to 4 weeks after the psilocybin session 2. In the Delayed Treatment group week 8 is a pre-psilocybin assessment obtained during the delay period. Effect size (Cohen's d with 95% confidence interval (CI)) and p-values reflect the results of a two-sample t-test between groups at study week 8 (d=1.9 [95% CI: 0.9; 2.8]).

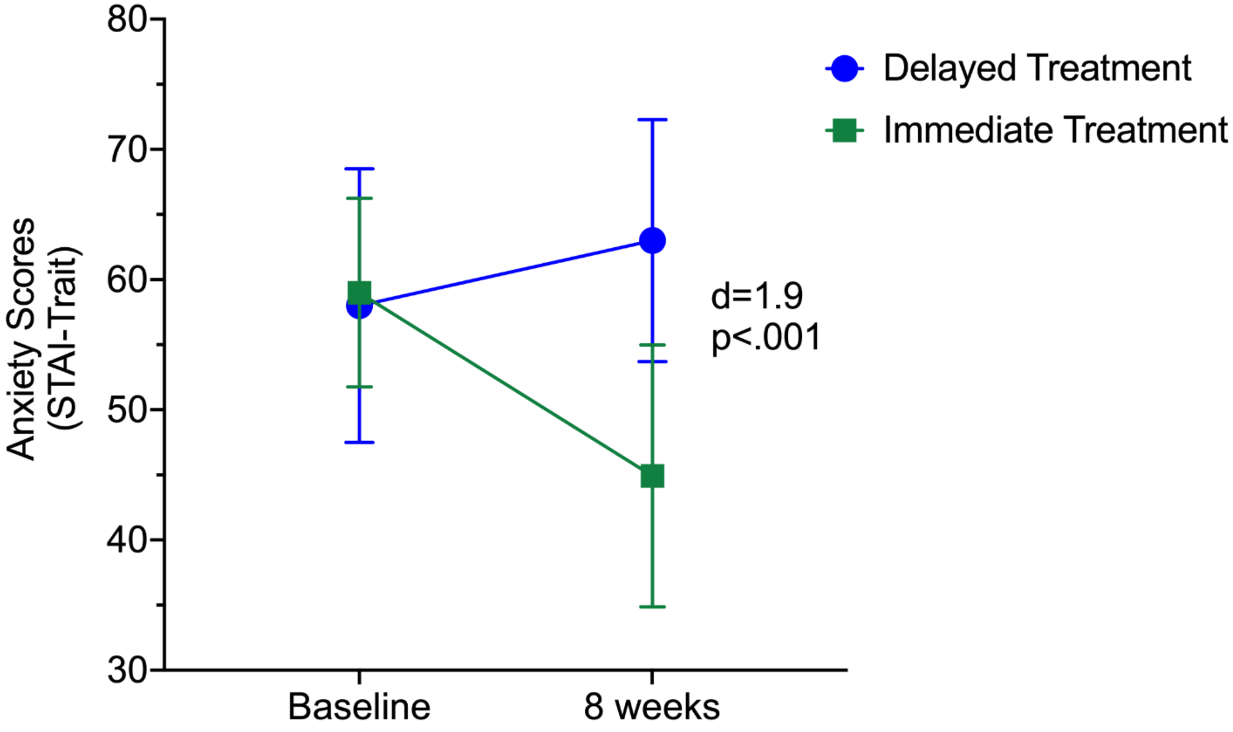

©2020 Davis AK et al. *JAMA Psychiatry*.

eFigure 8. Comparison of anxiety scores on the State-Trait Anxiety Inventory–Total Scale (STAI-Total) for the Delayed Treatment (n=11) and Immediate Treatment (n=13) conditions. Data points are means; brackets show ±1 SD. There were no missing data. Data are shown for Baseline (Screening) and study week 8. In the Immediate Treatment condition, study weeks 8 corresponds to 4 weeks after the psilocybin session 2. In the Delayed Treatment group week 8 is a pre-psilocybin assessment obtained during the delay period. Effect size (Cohen's d with 95% confidence interval (CI)) and p-values reflect the results of a two-sample t-test between groups at study week 8 (d=2.6 [95% CI: 1.5; 3.7]).

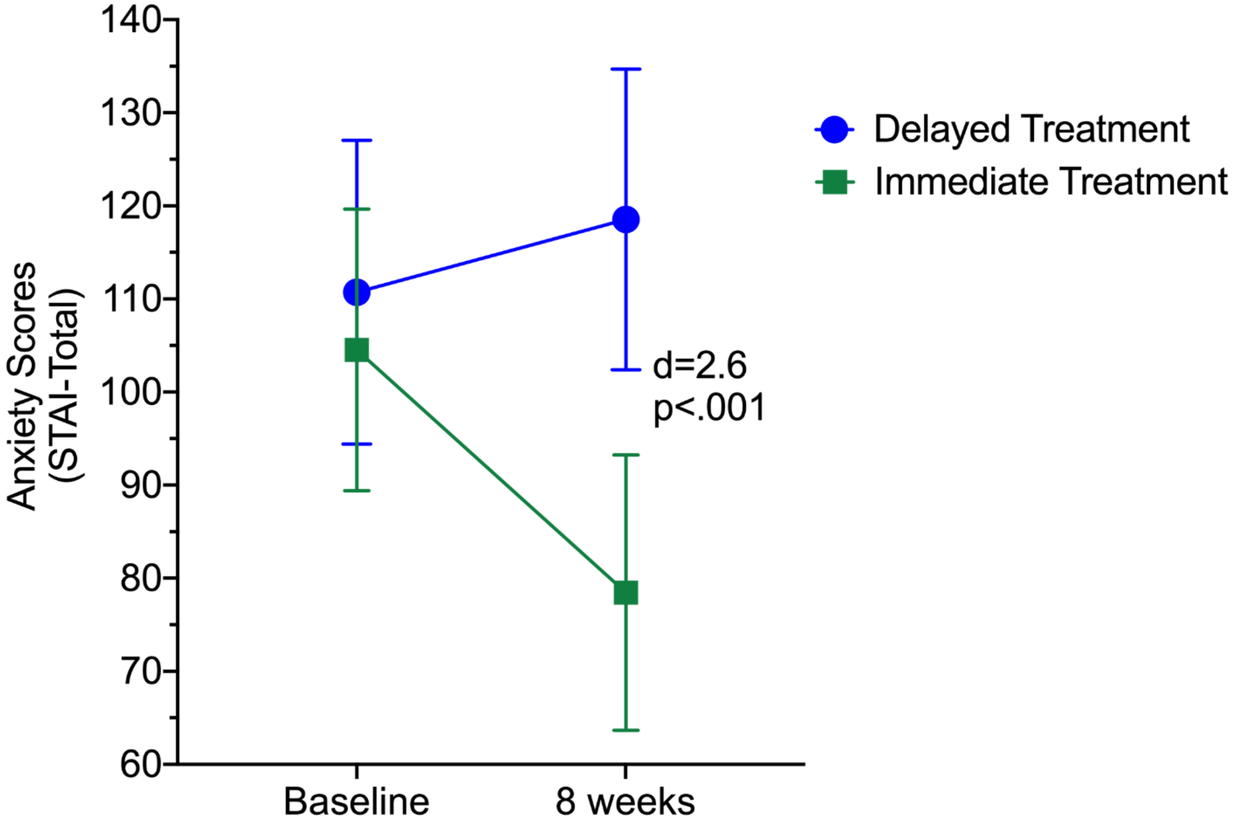

©2020 Davis AK et al. *JAMA Psychiatry*.

eFigure 9. Comparison of suicidal ideation scores on the Columbia Suicide Severity Rating Scale (CSSRS) for the Delayed Treatment (n=11) and Immediate Treatment (n=13) conditions. There were no missing data. Data points are means; brackets show + or - 1 SD. Data are shown for Baseline (Screening) and study week 8. In the Immediate Treatment condition, study weeks 8 corresponds to 4 weeks after the psilocybin session 2. In the Delayed Treatment group week 8 is a pre-psilocybin assessment obtained during the delay period.

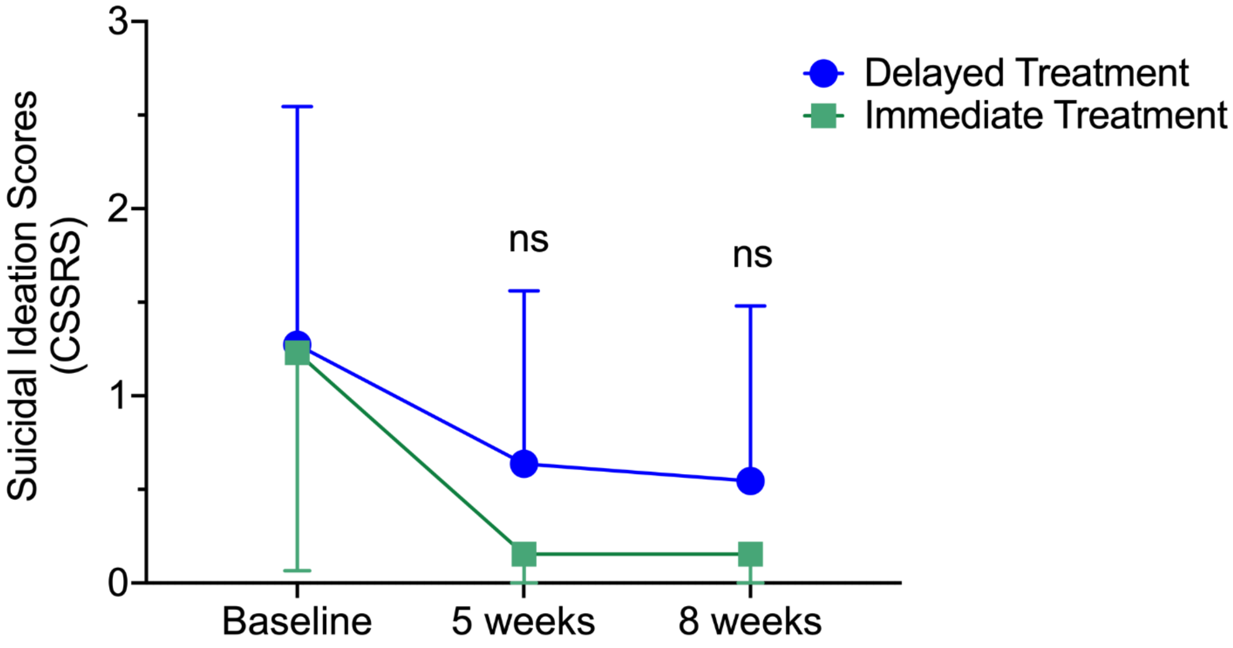

©2020 Davis AK et al. *JAMA Psychiatry*.
