# Supplementary material for: Effects of Psilocybin-Assisted Therapy on Major Depressive Disorder: A Randomized Clinical Trial
Source: JAMA Psychiatry. 2020 Nov 4;78(5):1–9. doi: 10.1001/jamapsychiatry.2020.3285 (PMC7643046; doi:10.1001/jamapsychiatry.2020.3285)
Supplement: Supplement 3. — Data sharing statement [file jamapsychiatry-e203285-s003.pdf]

Data Sharing Statement

Davis. Effects of Psilocybin-Assisted Therapy on Major Depressive Disorder. *JAMA Psychiatry*. Published November 04, 2020. 10.1001/jamapsychiatry.2020.3285

Data
Data available: No

Additional Information
Explanation for why data not available: The trial is ongoing to collect long-term follow-up data from trial participants. Data will be made available upon request to the corresponding author.
